# A Conserved Dopamine-Cholecystokinin Signaling Pathway Shapes Context–Dependent *Caenorhabditis elegans* Behavior

**DOI:** 10.1371/journal.pgen.1004584

**Published:** 2014-08-28

**Authors:** Raja Bhattacharya, Denis Touroutine, Belinda Barbagallo, Jason Climer, Christopher M. Lambert, Christopher M. Clark, Mark J. Alkema, Michael M. Francis

**Affiliations:** Department of Neurobiology, University of Massachusetts Medical School, Worcester, Massachusetts, United States of America; KU Leuven, Belgium

## Abstract

An organism's ability to thrive in changing environmental conditions requires the capacity for making flexible behavioral responses. Here we show that, in the nematode *Caenorhabditis elegans*, foraging responses to changes in food availability require *nlp-12*, a homolog of the mammalian neuropeptide cholecystokinin (CCK). *nlp-12* expression is limited to a single interneuron (DVA) that is postsynaptic to dopaminergic neurons involved in food-sensing, and presynaptic to locomotory control neurons. NLP-12 release from DVA is regulated through the D1-like dopamine receptor DOP-1, and both *nlp-12* and *dop-1* are required for normal local food searching responses. *nlp-12*/CCK overexpression recapitulates characteristics of local food searching, and DVA ablation or mutations disrupting muscle acetylcholine receptor function attenuate these effects. Conversely, *nlp-12* deletion reverses behavioral and functional changes associated with genetically enhanced muscle acetylcholine receptor activity. Thus, our data suggest that dopamine-mediated sensory information about food availability shapes foraging in a context-dependent manner through peptide modulation of locomotory output.

## Author Summary

Animal behavior is profoundly affected by contextual information about the internal state of the organism as well as sensory information about the external environment. A class of signaling molecules known as neuropeptides have been implicated in driving transitions between behavioral states (e.g., from food seeking to satiety and back) but we have only a limited understanding of how neuropeptide signaling modulates neural circuit activity and elicits context-dependent behaviors. Here we identify a novel mechanism by which *C. elegans* modulate their behavior in response to sensory information about food. We show that dopaminergic regulation of NLP-12, a *C. elegans* homolog of the mammalian neuropeptide cholecystokinin (CCK), shapes behavioral transitions that are central to food searching. Given the conserved nature of these signaling pathways, our work raises the interesting possibility that dopamine modulation of CCK signaling represents a general mechanism by which nervous systems shape context-dependent behavioral changes.

## Introduction

Animals have a remarkable capacity for altering their behavior in response to changes in both their external environment and their internal physiological state. Such behavioral modulation is often achieved through the actions of neuropeptides. Neuropeptides often act by modulating the effects of fast synaptic signaling in order to alter neuronal excitability and neural circuit activity. Striking examples of this have come from pioneering studies of rhythmic motor activity underlying feeding in crustaceans where neuropeptides and other neuromodulators potently alter neural activity patterns [1], [2]. The functional effects of specific neuromodulators can vary widely depending on levels of activity in target neurons as well as activity-dependent regulation of release, suggesting context-dependent modulation of circuit activity may enable flexible behavioral responses [3]–[5].

Local changes in food availability are among the most variable and significant environmental conditions that animals must cope with. Thus, mechanisms that regulate foraging behavior based on the availability of food are particularly important for survival. Recent studies using genetic approaches in worms, flies and mice have provided compelling evidence for state-dependent modulation of feeding and food-searching behavior [6]–[12]. In particular, these studies have elegantly demonstrated how neuromodulators signal information about internal factors, such as feeding state, and modulate sensory responsiveness to gustatory and olfactory stimuli. In contrast, we know relatively little about how sensory information signaling food availability is translated by the nervous system into alternative motor outputs such as those that underlie food searching.

The nematode *Caenorhabditis elegans* provides an attractive system to address neuromodulatory mechanisms involved in generating context-dependent behaviors. *C. elegans* exhibit robust behavioral responses to changes in their environment and we have a growing understanding of the sensorimotor circuits involved. In particular, changes in food availability alter *C. elegans* movement via effects on both locomotion velocity and turning frequency. Most strikingly, removal of *C. elegans* from food initiates an alternative motor pattern in which animals restrict movement to their immediate environment. This behavior shares many features with a local foraging strategy, known as area-restricted search [13]–[16] that is observed across almost all animal species. Following removal from food *C. elegans* transiently increase their turning frequency and then, if unsuccessful in finding food, shift within minutes to longer runs of uninterrupted forward movement in order to disperse over larger areas. Thus, *C. elegans* movement is potently affected by external information about the availability of food in the local environment, and sensory information about food availability drives context-dependent behavioral transitions that are central to foraging.

To elucidate mechanisms responsible for generating context-dependent modulation of behavior, we used a genetic strategy to identify candidate neuropeptides and assessed their role in modulation of *C. elegans* movement. We observed that dopaminergic regulation of neuropeptide signaling plays a central role in shaping context-dependent motor responses, such as those that underlie local food searching. These effects are mediated through the actions of the NLP-12 neuropeptide, a *C. elegans* homolog of mammalian cholecystokinin (CCK). Under normal (well-fed) conditions, *nlp-12* is not required for exploratory movement; however, a behavioral requirement for NLP-12 signaling is revealed by a genetic manipulation that produces increased synaptic activation of muscles. Release of NLP-12, which is solely expressed in the interneuron DVA, is modulated by the D1-like dopamine receptor DOP-1 and both *dop-1* and *nlp-12* are required for normal foraging responses to food deprivation. While *nlp-12* deletion impairs foraging through reductions in body bend depth and turning, *nlp-12* overexpression is sufficient to stimulate deep body bends and enhance turning, and these effects require wild type acetylcholine receptor function in muscles. Thus, NLP-12/CCK shapes motor circuit responsiveness to sensory information through context-dependent modulation of locomotory output.

## Results

### Enhancing L-AChR function promotes deep body bends during movement

**Figure 1:**
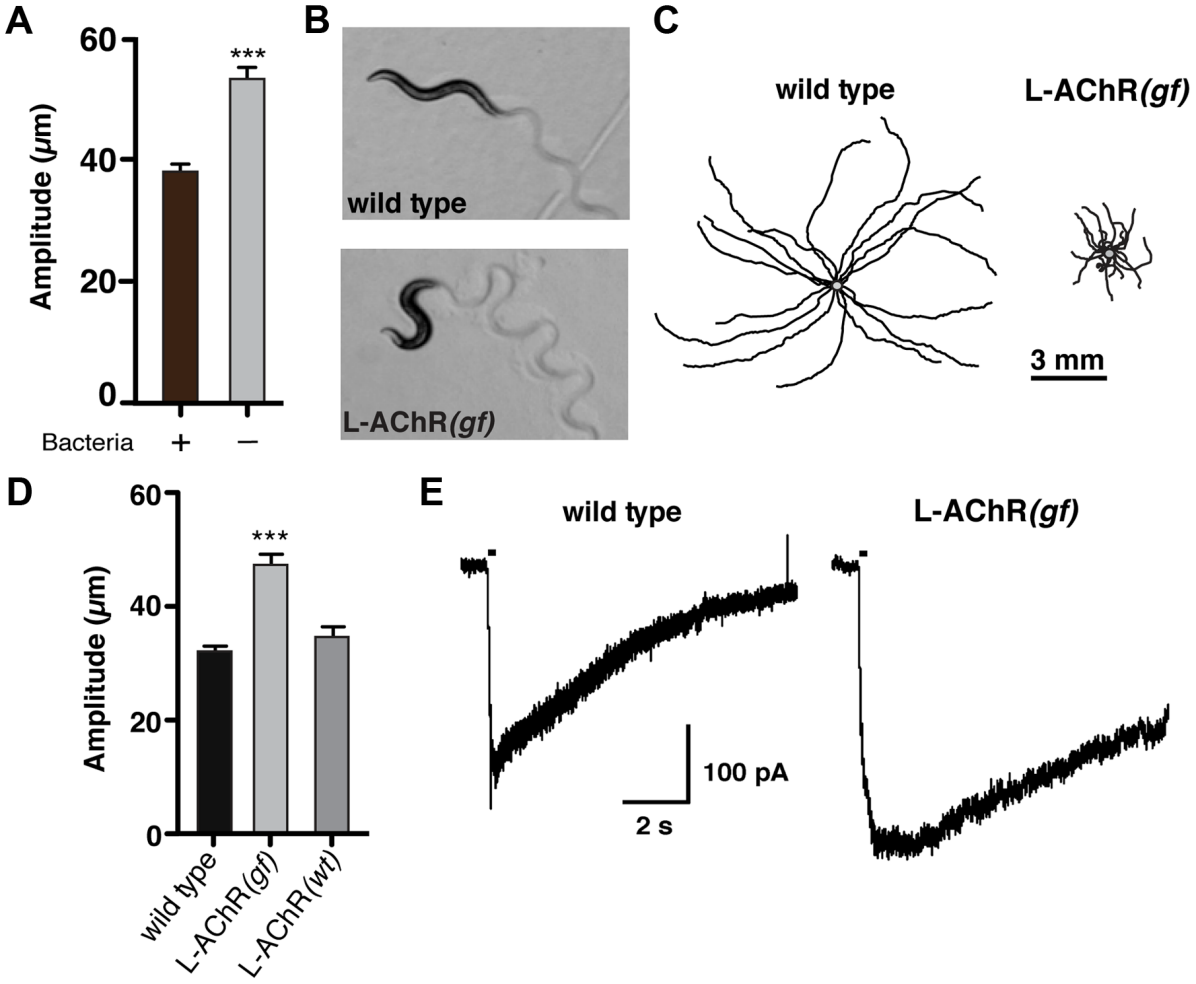
Enhanced L-AChR function increases neuromuscular signaling and alters the *C. elegans* locomotory pattern. (**A**) Average body bend amplitude of wild type worms in presence (+) or absence (−) of bacterial food. Well-fed animals were transferred to assay plates with or without food and videotaped for 45 s following a recovery period of one minute. Bars represent the mean (±SEM) of values calculated from 15 animals. ***, p<0.0001 student's t-test. (**B**) Representative images of wild type and L-AChR*(gf)* animals. Note the exaggerated track amplitudes and the hypercontracted body posture of L-AChR*(gf)* animals. (**C**) Movement trajectories of wild type and L-AChR*(gf)* animals. Each black line shows the trajectory of one animal monitored for 45 s on food. In this and subsequent figures, tracks are aligned to a common center point (gray) for clarity. (**D**) Average body bend amplitude for wild type, L-AChR*(gf)* and L-AChR*(wt)* animals as indicated. Values for body bend amplitude were calculated from recordings of the tracks shown in C. Bars represent the mean (±SEM) of values calculated from at least 15 animals. ***, p<0.0001 by ANOVA and Sidak's post-hoc test. (**E**) Current responses to pressure application of levamisole (100 µM) recorded from body wall muscles of wild type and L-AChR*(gf)* animals. Holding potential was −60 mV. Black bars indicate the duration of levamisole application (200 ms). See Figure 6 for additional electrophysiological characterization of the L-AChR*(gf)* strain.

*C. elegans* moves sinusoidally by propagating waves of dorsoventral flexures along the length of its body. The pattern of *C. elegans* movement is profoundly affected by information from the environment. For example, mechanosensory stimuli such as those associated with mating and predation, or changes in food availability can each evoke alternative motor outputs. To precisely define how sensory stimuli conveying food availability affects sinusoidal movement, we monitored body bends immediately following removal from food using an automated tracking system. We found that removal of *C. elegans* from food produced a significant increase in body bend depth (40±5% increase, p<0.0001) (Fig. 1A), suggesting that active modulation of body bends is an important component of *C. elegans* motor responses to changes in food availability. To further investigate how sensory perception of food may be translated into altered motor responses, we sought to develop a genetic strategy that would mimic aspects of the motor responses to food removal. We hypothesized that modulation of body bend amplitude may be achieved through increased synaptic activation of body wall musculature. To gain support for this idea, we used a genetic approach to enhance the activity of neuromuscular synapses.

To increase neuromuscular synapse activity, we expressed an engineered form of a muscle ionotropic acetylcholine receptor (iAChR) with enhanced function. In *C. elegans*, two classes of iAChRs – levamisole sensitive (L-AChR) and nicotine sensitive (N-AChR) are expressed in muscle cells [17]–[19]. L-AChRs are heteropentameric complexes composed of the subunits UNC-38, UNC-63, LEV-1, LEV-8 and UNC-29 [20]. Loss of L-AChR function leads to a characteristic reduction of cholinergic currents recorded from muscles and sluggish movement [18], [21]–[23]. To determine if we could enhance synaptic activation of muscles by increasing L-AChR function, we introduced an amino acid change at a highly conserved position in the pore-lining M2 regions of cDNAs encoding the UNC-29, UNC-38 and LEV-1 subunits (Fig. S1A). Substitution of a polar amino acid (*e.g.* serine) for the native residue at this position (typically leucine) produces increased activation of mammalian iAChRs [24]–[27]. Specific expression of individual pore-modified L-AChR subunits in muscles led to phenotypic changes consistent with increased muscle excitability (*e.g.* altered movement). To enhance this effect, we constructed a strain (*ufIs6*) stably expressing the *unc-29*(L/S), *unc-38*(V/S) and *lev-1*(L/S) cDNAs under control of the muscle-specific *myo-3* promoter, allowing for incorporation of 3 pore-modified subunits into each pentameric receptor complex. Hereafter, we refer to this transgenic strain as L-AChR(*gf*). Visual analysis of muscle structure and neuromuscular synapses indicated L-AChR(*gf*) expression did not produce obvious muscle toxicity or disruption of neuromuscular synapse development (Fig. S1B, C). Moreover, animals expressing L-AChR(*gf*) receptors were hypersensitive to the paralyzing effects of the L-AChR specific ligand levamisole, indicating this manipulation produced the predicted activating effects on the receptor (Fig. S2A, B).

To quantify behavioral changes produced by L-AChR(*gf*) expression, we monitored the exploratory movements of transgenic animals. L-AChR(*gf*) expression produced a significant increase in body bend amplitude compared to the wild type (48.3±5% increase, p<0.0001), while movement velocity was decreased (Fig. 1B–D and Fig. S2C). These results suggested L-AChR(*gf*) expression enhanced muscle contractions in response to synaptic ACh release from motor neurons, altering the sinusoidal motor pattern. Consistent with this, L-AChR(*gf*) animals were shorter in body length compared to the wild type, likely as a consequence of muscle hypercontraction (Fig. S2D). In contrast, an integrated transgene (*ufIs47*) encoding wild type copies of the same L-AChR subunits under control of a muscle-specific promoter did not produce strong effects on any of the measures we made, supporting the notion that the behavioral changes were caused by enhanced function of the engineered receptor. Finally, electrophysiological recordings from body wall muscles demonstrated that L-AChR(*gf*) expression prolonged the duration of current responses to levamisole, providing direct evidence that the engineered mutation altered muscle synapse activity by altering receptor functional properties (Fig. 1E). Thus, genetically increasing synaptic excitation of muscles is sufficient to produce dramatic increases in body bend depth.

### NLP-12/CCK neuropeptide is required for locomotor effects of enhanced muscle activation

**Figure 2:**
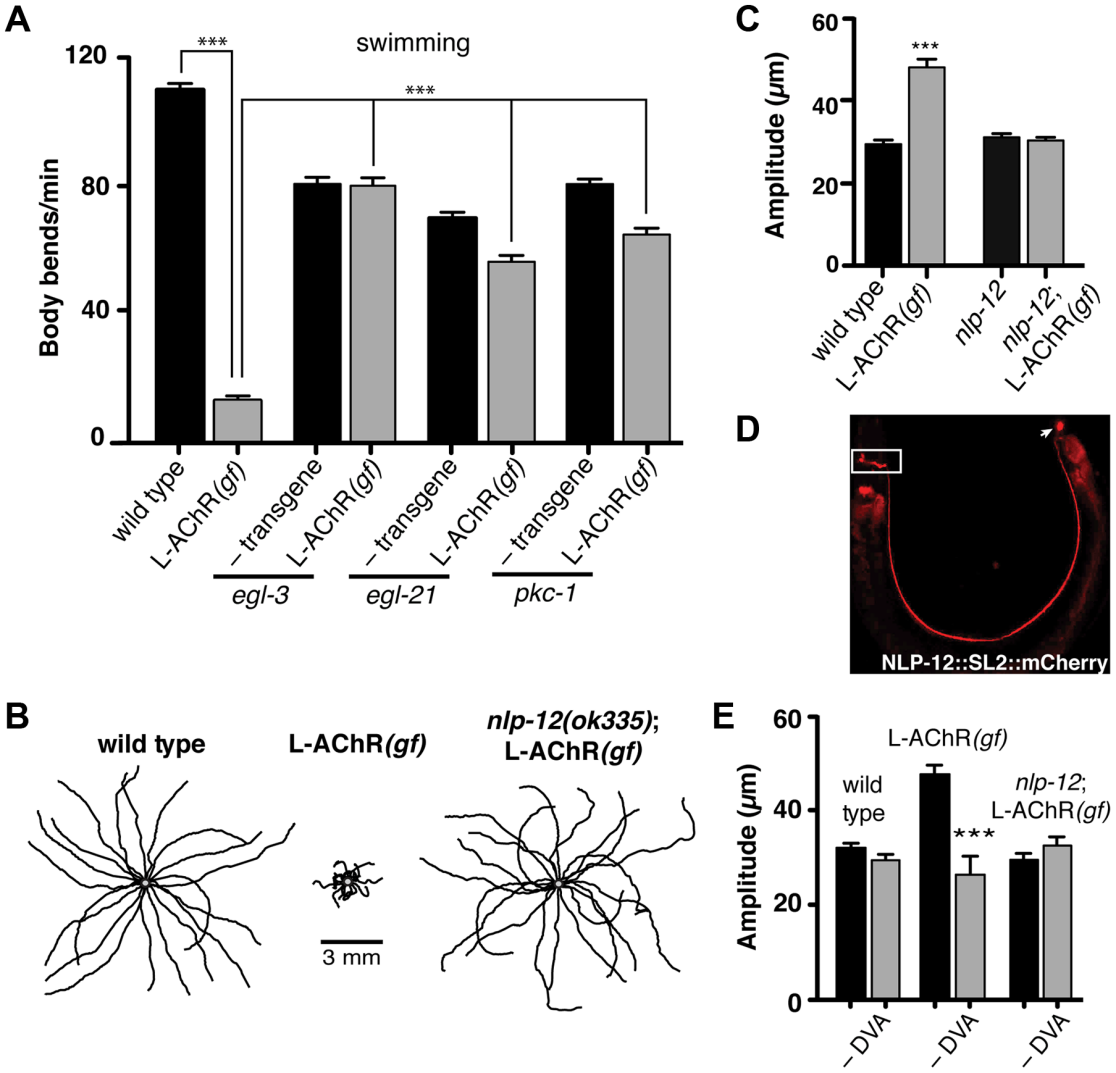
Locomotory phenotypes associated with L-AChR*(gf)* expression require neuropeptide signaling. (**A**) Average body bends/min measured in liquid for the genotypes indicated. The strong locomotory defects of *egl-3* and *egl-21* mutants prevented analysis of L-AChR(*gf*) effects on agar (Fig. S3A, B). Mutation of *pkc-1* normalized the locomotor effects of L-AChR(*gf*) in both liquid and on agar (Fig. S3C–E). (**B**) Movement trajectories of wild type, L-AChR*(gf), nlp-12*;L-AChR*(gf)* animals as indicated. Each black line shows the trajectory of one animal monitored for 45 s on food. (**C**) Average body bend amplitude for the genotypes indicated. Each bar represents the mean (±SEM) of values calculated from recordings of at least 15 animals. For (A) and (C) ***, p<0.0001 by ANOVA with Sidak's post-hoc test. (**D**) Wide-field epifluorescent image of an adult animal expressing *nlp-12::SL2::mCherry*. The image is oriented with the head to the left. White rectangle: nerve ring. Arrow: DVA cell body. (**E**) Average body bend amplitude for the indicated genotypes and effects of DVA ablation (–DVA). Ablations were performed on L2 stage animals. Body bend amplitude was measured from recordings of young adult animals 2 days following laser ablation. Error bars indicate mean (±SEM) of at least 8 animals. ***, p<0.0001 student's t-test.

The above results suggested that analysis of the alternative motor pattern produced by increased neuromuscular synapse activation might provide insights into similar motor patterns that occur during foraging behaviors. As neuropeptides and other neuromodulators often play instrumental roles in generating flexible behavioral responses [1], [28], we next investigated if loss-of-function mutations in the neuropeptide processing enzymes *egl-3*/PC2 proprotein convertase or *egl-21*/carboxypeptidase E [29]–[33] might modulate the behavioral effects of L-AChR(*gf*) expression. The extremely sluggish movement of either *egl-3* or *egl-21* mutants in the absence of L-AChR(*gf*) expression hindered our efforts to address this question on solid agar plates (Fig. S3). In contrast, we found that mutations in *egl-3* and *egl-21* caused more modest locomotory defects in a related assay that assesses movement in liquid (swimming), enabling analysis of L-AChR(*gf*) effects. Transgenic L-AChR(*gf*) animals were significantly less active than either wild type animals or the neuropeptide-deficient mutants. Further, we observed that mutation of either *egl-3* or *egl-21* suppressed the effects of L-AChR(*gf*) expression, improving movement (Fig. 2A). While neurotransmitters involved in fast synaptic transmission are packaged into small clear synaptic vesicles, neuropeptide signaling is mediated through release of neuropeptide-laden dense core vesicles (DCV). To gain further support for neuropeptide involvement, we analyzed mutations in the *pkc-1* gene. *pkc-1* encodes a member of the novel Protein Kinase C family (nPKC) most closely related to vertebrate nPKCε/η [34], and was previously implicated in the regulation of DCV release without obvious effects on fast synaptic transmission at the NMJ [35]. Mutation of *pkc-1* caused only modest effects on movement in otherwise wild type animals; yet, it reversed L-AChR(*gf*)-mediated behavioral changes almost completely (Figs. 2A and S3). Thus, normal neuropeptide processing and secretion is required for L-AChR(*gf*) effects on movement. Together, our findings suggest that neuropeptide signaling modulates locomotory behaviors associated with high neuromuscular synapse activity.

Loss of either EGL-3 or EGL-21 function impacts biosynthesis of a variety of peptides, raising the question of which peptides play primary roles in modulating neuromuscular signaling and movement. To address this question, we undertook a suppressor approach, using RNAi to knockdown expression of candidate neuropeptide precursors in animals expressing the L-AChR(*gf*) transgene. Sequence analysis of the *C. elegans* genome predicts more than 100 candidate neuropeptide precursors including insulin-like (*ins*), FMRFamide-like (*flp*) and neuropeptide-like (*nlp*) protein family members [36]. In total, we targeted 66 of these precursors spanning each of the 3 gene families. Remarkably, downregulating the neuropeptide-like *nlp-12* precursor, a *C. elegans* homolog of cholecystokinin produced dramatic effects. RNAi targeting *nlp-12* increased the movement velocity of transgenic L-AChR(*gf*) animals by more than 4-fold (L-AChR(*gf*) empty vector: 12.7±2 body bends/min, L-AChR(*gf*) *nlp-12* RNAi: 49±3 body bends/min, p<0.0001) to a level that was indistinguishable from that of wild type animals (WT: 41.5±2 body bends/min). In contrast, RNAi targeting *nlp-12* in wild type animals had no obvious effects on movement [in the presence of bacterial food] (not shown). To further support a role for *nlp-12* in modulating locomotory behaviors, we analyzed the effects of L-AChR(*gf*) expression in a strain carrying a deletion mutation (*ok335*) in the *nlp-12* locus. *nlp-12* gives rise to 2 distinct mature peptides that share similarity with mammalian cholecystokinin-8, the predominant form of CCK in mammalian neurons [37], [38]. The *ok335* allele corresponds to a 1070 bp deletion that disrupts both of the mature NLP-12 peptides. We measured body bend amplitude, movement velocity and body length in *nlp-12* mutants carrying the L-AChR(*gf*) transgene and found that each of these measures was brought to near wild type levels by deletion of *nlp-12* (Fig. 2B, C and Fig. S4A, B). Similarly, *nlp-12* deletion reversed the effects of L-AChR(*gf*) expression in liquid movement assays (Fig. S4C). Surprisingly, while loss of *nlp-12* function suppressed the effects of L-AChR(*gf*) in locomotion, no locomotion defect was apparent in these animals in the absence of L-AChR(*gf*) (under our normal growth conditions). This finding is consistent with the notion that NLP-12 signaling may be preferentially required during certain behavioral states, a hypothesis we tested below.

### The interneuron DVA is required for NLP-12/CCK modulation of movement

To further define *nlp-12* effects, we examined the expression of a reporter construct containing the native NLP-12 genomic sequence SL2 trans-spliced to the fluorescent reporter mCherry. Consistent with previous studies [37], [39], fluorescence was solely visible in DVA and was present from early embryonic stage (not shown) throughout adulthood (Fig. 2D). DVA is an interneuron that relays mechanosensory information onto the motor circuit through synaptic outputs onto both premotor interneurons and motor neurons [40], [41]. The DVA cell body is located in the dorsorectal ganglia of the tail and extends a single process along the length of the ventral nerve cord into the nerve ring. The restricted expression of *nlp-12* to DVA suggested an essential role for this neuron in NLP-12 modulation of the motor pattern. We tested this idea in laser ablation experiments. DVA ablation in L-AChR(*gf*) animals normalized body bend amplitude (Fig. 2E) and movement velocity (Fig. S4B). In contrast, ablation of DVA caused no obvious effects in either *nlp-12*;L-AChR(*gf*) or wild type animals under normal food conditions (Fig. 2E and [42], [43]). Blocking vesicular release from DVA by DVA-specific expression of Tetanus toxin (Tetx) produced similar effects (Fig. S4D). Thus, *nlp-12* deletion, cell-specific DVA ablation or block of vesicular release from DVA, the only neuron with detectable levels of *nlp-12* expression, each similarly reversed the effects of enhanced muscle excitation. Prior work has suggested that one function for DVA and NLP-12 signaling may be to convey proprioceptive information that is signaled through stretching associated with muscle contraction and movement [39], [40]. Our results support the model that NLP-12 effects are mediated via secretion from DVA, and demonstrate a behavioral requirement for NLP-12 and DVA with increased muscle synapse activity.

### NLP-12/CCK regulates foraging behavior

**Figure 3:**
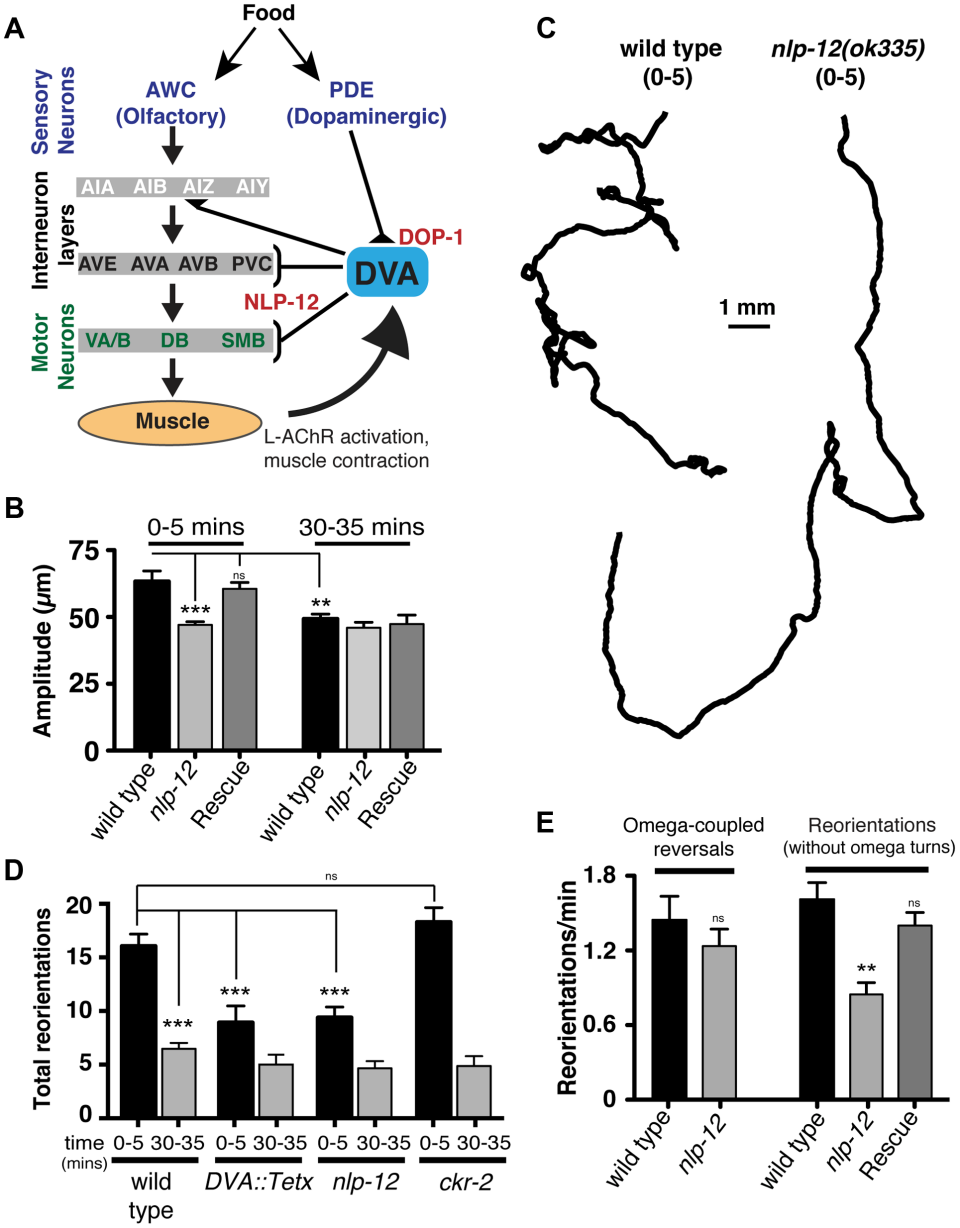
Modulation of body bend depth through NLP-12/CCK signaling is critical for local food searching. (**A**) Schematic representation of the neural circuit underlying NLP-12 modulation of local searching. Synaptic connections (triangles, brackets) are as described by [42]. DVA receives synaptic input from the dopaminergic neuron PDE and makes connections with both motor neurons and interneurons involved in locomotory control. DVA makes synaptic contacts onto all of the motor and interneurons indicated by brackets. In addition, DVA is connected to AVB and PVC by gap junctions. Assignments of interneurons into layers are as described by [13]. Other neuron classes are as described in the text and references therein. DOP-1 modulation of DVA activity regulates NLP-12 release from DVA, altering the motor pattern during local food searching. (**B**) Average body bend amplitude for the genotypes as indicated measured during an initial five minute time interval (0–5) immediately following removal from food and a second five minute time interval 30 minutes after removal from food (30–35). Bars represent mean values (±SEM) calculated from 9–14 animals. (**C**) Representative tracks of wild type and *nlp-12(ok335)* mutants during an initial five minute period (0–5) following removal from food. Note the decreased number of reorientations and increased frequency of long forward runs for *nlp-12(ok335)* mutant as compared to the wild type. (**D**) Total directional reorientations measured during the 0–5 and 30–35 minute intervals following removal from food for wild type, transgenic wild type animals expressing Tetanus toxin in DVA *[DVA::Tetx]*, *nlp-12(ok335)* and *ckr-2(tm3082)* mutants. Bars represent mean (±SEM) for at least 12 animals. (**E**) Quantification of reversal coupled omega turns and reorientations in the absence of omega turns during the first 5 minutes following removal from food for the genotypes indicated. (WT and *nlp-12(ok335)*: n = 17). Rescue refers to the *nlp-12(ok335)* mutant expressing an extrachromosomal array carrying *Pnlp-12::nlp-12::SL2::mCherry* (n = 9). Bars represent mean (±SEM). ***, p<0.0001; **, p<0.001 by ANOVA with Sidak's post-hoc test.

Our experiments provided evidence for NLP-12 modulation of the motor pattern when muscle excitability was altered genetically; however, under normal growth conditions, DVA ablation or *nlp-12* deletion had only minimal effects on exploratory movement. These observations suggest NLP-12 signaling may be preferentially active during particular behaviors. A major source of synaptic input to DVA comes from dopaminergic mechanosensory neurons (PDE) that contribute to food sensing [44], [45] (Fig. 3A). This pattern of connectivity suggests DVA may integrate proprioceptive information with sensory information from PDE. Our earlier behavioral analysis showed wild type animals deepen their body bends following removal from food (Fig. 1A). To evaluate whether NLP-12 signaling may contribute to modulation of body bend amplitude during these responses, we investigated the effects of *nlp-12* deletion in the context of food deprivation (Fig. 3B). Interestingly, *nlp-12* deletion significantly reduced body bend depth in food-deprived wild type animals (25±1% reduction, p<0.01). Normal body bend modulation upon food deprivation was restored by expression of *nlp-12* under the native promoter. These results indicate that NLP-12 modulation enables deepening of body bends in response to sensory information about food availability.

During our analysis of wild type behavior, we noted that body bend depth was initially increased following removal from food but then normalized with increasing time off food. To quantify this effect, we compared the depth of body bends during an initial 5-minute period following removal from food to that measured 30 minutes later. We found that body bend depth was significantly reduced 30 minutes after removal from food (22.2±3% reduction, p<0.01) (Fig. 3B). Removal of *C. elegans* from food induces an immediate switch to an alternative local searching motor pattern, often referred to as area-restricted search [13]–[16]. During this motor pattern, *C. elegans* increase the frequency of reorientations in their direction of movement (high-angle turns), an effect thought to represent a strategy for localized searching. With increased time following removal from food, these reorientations decrease in frequency, allowing for longer runs of forward movement and dispersal of the animals. The transient increase in body bend depth we observed after removing wild type animals from food suggested that modulation of body bend depth might contribute to local food searching behavior. To investigate this possibility, we monitored movement during a 35 minute period immediately following removal from food and quantified reversals and turning behavior during the first (0–5) and last (30–35) five minutes (Fig. 3C–E). Wild type animals displayed frequent, large reorientations in their movement trajectory during an initial five-minute time period immediately following removal from food (0–5 min: 16.1±1.1 reorientations), consistent with previous descriptions of local searching behavior [13], [14]. By 30 minutes after removal from food, the movement pattern of wild type animals shifted to longer runs and the number of high angle turns (30–35 min: 6.5±0.6 reorientations) decreased significantly from those measured during the initial five minute period of food deprivation (60±3% decrease, p<0.0001) (Fig. 3D). Specific expression of TetX in DVA or *nlp-12* deletion significantly reduced the frequency of high-angle reorientations during the initial five-minute period following removal from food (43±9% decrease and 42±6% decrease respectively, p<0.0001) (Fig. 3C, D). These results suggest that the local search motor pattern is dependent on DVA and NLP-12 signaling.

Neuropeptides generally act through G-protein coupled receptors (GPCRs). NLP-12 directly binds the GPCR Cholecystokinin-like Receptor 2 (CKR-2) *in vitro* and previously described roles for *nlp-12* have indicated a requirement for *ckr-2* [37], [39]. Surprisingly however, we found that *ckr-2* was not required for normal local food searching behavior (Fig. 3D). This result suggests an interesting possibility that NLP-12 modulation of foraging behavior relies on additional GPCR signaling pathways which operate independently or in parallel with CKR-2. There are several genes encoding GPCRs with significant homology to *ckr-2* in the worm genome (*e.g. ckr-1*, *npr-2*, *npr-5*). While NPR-5 is activated by the neuropeptide FLP-18 when expressed in *Xenopus* oocytes [46], CKR-1 and NPR-2 remain uncharacterized, so these genes are good candidates for additional components of the NLP-12 signaling pathway.

Reorientations during local searching occur primarily through 2 mechanisms: reversals coupled to sharp, ventrally directed head-to-tail turns (referred to as omega turns), or large head swings accompanied by deep body bends that establish a new heading [47], [48]. For wild type animals, approximately 50% of the trajectory changes we observed were initiated by omega turns. Interestingly, the frequency of omega turns was not significantly decreased by *nlp-12* deletion (Fig. 3E). Instead, we found that deletion of *nlp-12* caused a significant reduction in the frequency of trajectory changes that were not associated with omega turns (47±6% decrease, p<0.0001). This reduction was rescued by *nlp-12* expression under the native promoter. Previous work has demonstrated that olfactory information about food availability shapes local searching primarily by increasing the frequency of omega turning [13]. Our findings indicate that NLP-12 modulates local searching by affecting changes in trajectory that arise independently of omega turns. Thus, NLP-12-mediated locomotory changes during local searching are likely to be produced through a mechanism that is distinct from those elicited by changes in olfactory neuron activity.

### Dopamine stimulates NLP-12 release and shapes foraging behavior through the dopamine receptor DOP-1

**Figure 4:**
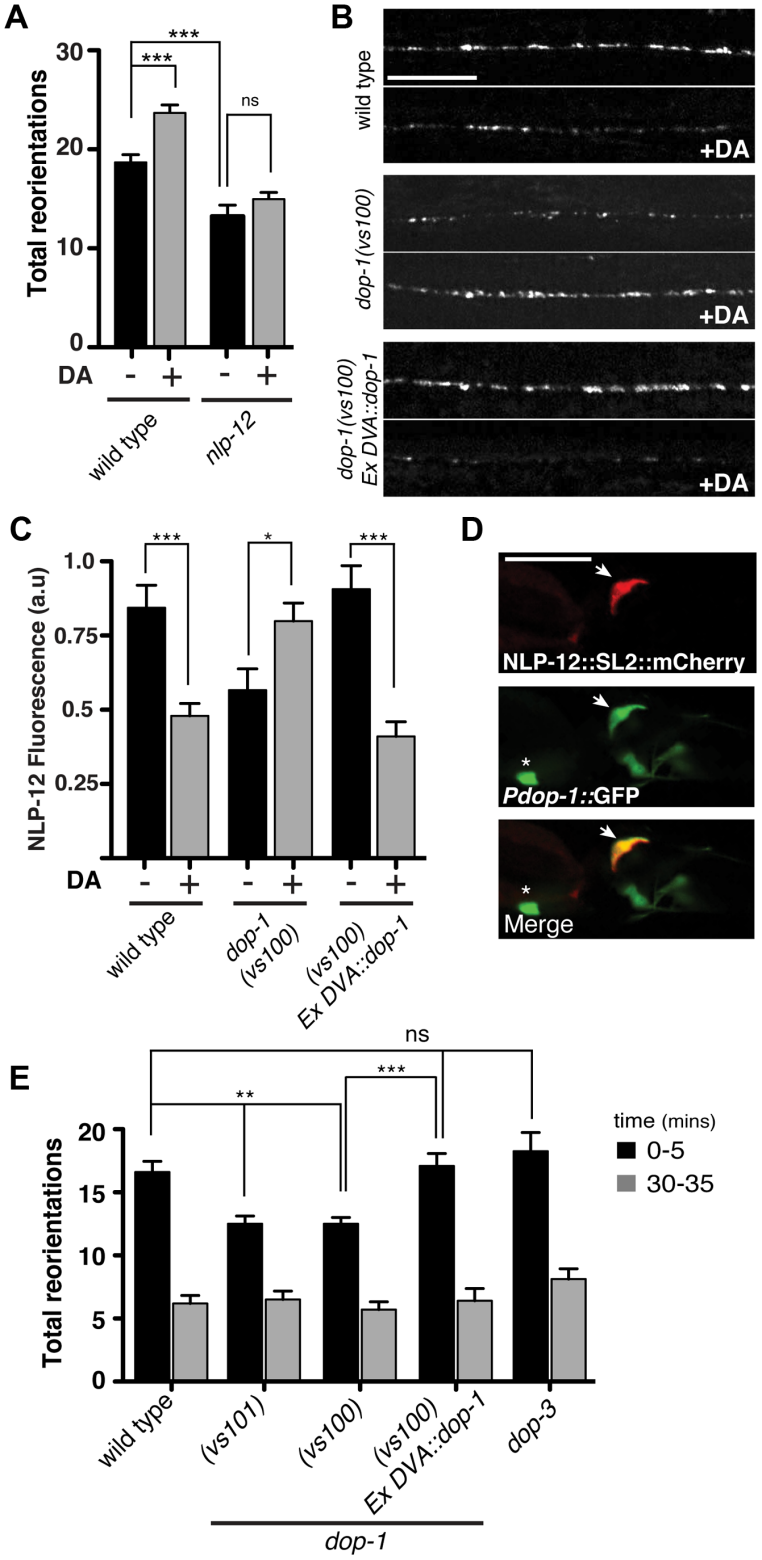
The dopamine receptor DOP-1 is required in DVA for NLP-12 modulation of food searching. (**A**) Frequency of high angled reorientations for wild type and *nlp-12(ok335)* animals quantified for 5 minutes after transfer to food free plates in the presence (+) or absence (−) of dopamine (DA). Bars represent mean (±SEM) for at least 12 animals. Dopamine mechanosensory signaling is strongly enhanced at low osmotic strength [73]. Therefore, these assays were conducted following transfer of the animals to low osmotic-strength assay plates as described previously [14]. We observed a modest increase in basal reorientation frequency across all genotypes under these conditions. (**B, C**) Representative images (B) and quantification (C) of NLP-12::VenusYFP fluorescence in the ventral cord region of the DVA process of wild type, *dop-1(vs100)*, and *dop-1(vs100) Ex DVA::dop-1* animals before (−) and after (+) 10 minutes dopamine (DA) treatment (wild type: n = 12 for (−) and (+) DA; *dop-1(vs100)*: n = 12 for (−) and 9 for (+) DA). *Ex DVA::dop-1* refers to specific rescue of *dop-1* expression in DVA using the *nlp-12* promoter (−DA, n = 12; +DA, n = 11). Bars represent mean ±SEM. ***, p<0.0005; *, p<0.05 student's t-test. (**D**) Single slice confocal images of the DVA neuron in a transgenic animal expressing *nlp-12::SL2::mCherry* (upper panel) together with *Pdop-1::GFP* (middle panel). White arrow denotes the DVA interneuron in all cases. Asterix denotes a ventral cord motor neuron expressing the *dop-1* reporter. Scale bars in B and D, 20 µm. (**E**) Total directional reorientations measured during 0–5 and 30–35 minute intervals following removal from food for the genotypes as indicated. WT: n = 10; *dop-1(vs101)*: n = 12, *dop-1(vs100)*: n = 14, *dop-1(vs100) Ex DVA::dop-1*: n = 12 and *dop-3(vs106)*: n = 8. Bars represent mean (±SEM). For (A) and (E) ***, p<0.0005, **, p<0.005 by ANOVA with Sidak's post-hoc test.

Our above behavioral experiments indicated that foraging responses to local changes in food availability were shaped by NLP-12 signaling. DVA receives strong synaptic projections from the dopaminergic PDE neurons and one proposed role for dopaminergic neurons is in food sensing [44], [45]. Therefore, we next investigated the effects of dopamine on foraging behavior. Initially, we examined whether exogenous dopamine was sufficient to alter the motor pattern of wild type animals and, consistent with previous work [14], found that a brief exposure to exogenous dopamine was sufficient to elicit increases in reorientations during movement (27±4% increase, p<0.01). The effects of dopamine required *nlp-12*, providing support for the notion that dopamine signaling may regulate NLP-12 release (Fig. 4A). Prior studies in *C. elegans* have used fluorescently labeled neuropeptide precursors to monitor neuropeptide release [35], [39]. Therefore to directly test the idea that dopamine signaling stimulates NLP-12 release, we expressed NLP-12-VenusYFP in DVA. In the absence of exogenous dopamine, we observed a punctate pattern of NLP-12-VenusYFP in the DVA process, consistent with prior work [39]. If dopamine signaling onto DVA leads to significant release of NLP-12, we would expect acute dopamine treatment to reduce punctate NLP-12 fluorescence in DVA. Consistent with this, we found brief exposure to dopamine produced a significant decrease in NLP-12-VenusYFP fluorescence (Fig. 4B, C) To gain support for the idea that this was a direct effect of dopamine on DVA, we examined dopamine receptor expression and found that a transcriptional reporter for the *dop-1* gene was strongly expressed in DVA (Fig. 4D). *dop-1* encodes a D1-like dopamine receptor previously implicated in modulation of sensory neuron responses to touch [49]–[51]. Acute exposure of *dop-1* mutants to dopamine did not produce significant decreases in NLP-12-VenusYFP fluorescence, suggesting that dopamine stimulation of NLP-12 release required DOP-1. In addition, we noted that mutation of *dop-1* produced decreased levels of basal fluorescence, suggesting DOP-1 also regulates unstimulated NLP-12 levels. Specific rescue of *dop-1* expression in DVA normalized basal NLP-12 levels and importantly, restored sensitivity to dopamine. Thus, our results support the notion that dopamine acts to stimulate NLP-12 release via direct activation of the D1-like receptor DOP-1 in DVA. To address whether DOP-1 stimulation of NLP-12 release played a central role in modulating foraging behavior, we examined the food-searching behavior of *dop-1* mutants. Prior work has demonstrated that mutations in *dop-1* alone do not impair exploratory movement in the presence of food [52]. We found that *dop-1* mutants were defective in foraging responses to food deprivation, behaving similarly to *nlp-12* mutants. Specifically, the frequency of high-angle reorientations following removal from food was significantly decreased in *dop-1* mutants compared to the wild type (25±3% decrease, p<0.001)(Fig. 4E). These effects were rescued by specific expression of wild type *dop-1* in DVA and were not observed with mutation of the related dopamine receptor *dop-3*. Conversely, behavioral changes elicited by increasing muscle synapse activation did not show a strong requirement for dopamine signaling (*e.g.* L-AChR(*gf*) body bend depth: 72±4 µm, *cat-2*;L-AChR(*gf*) body bend depth: 73.4±2 µm), suggesting that proprioceptive signaling and dopamine mechanosensory signaling can each independently affect DVA activity and NLP-12 release. Taken together, our results support a model where dopamine signaling through DOP-1 regulates foraging by direct modulation of DVA activity and NLP-12 release.

### Elevated NLP-12/CCK signaling enhances bending during movement

**Figure 5:**
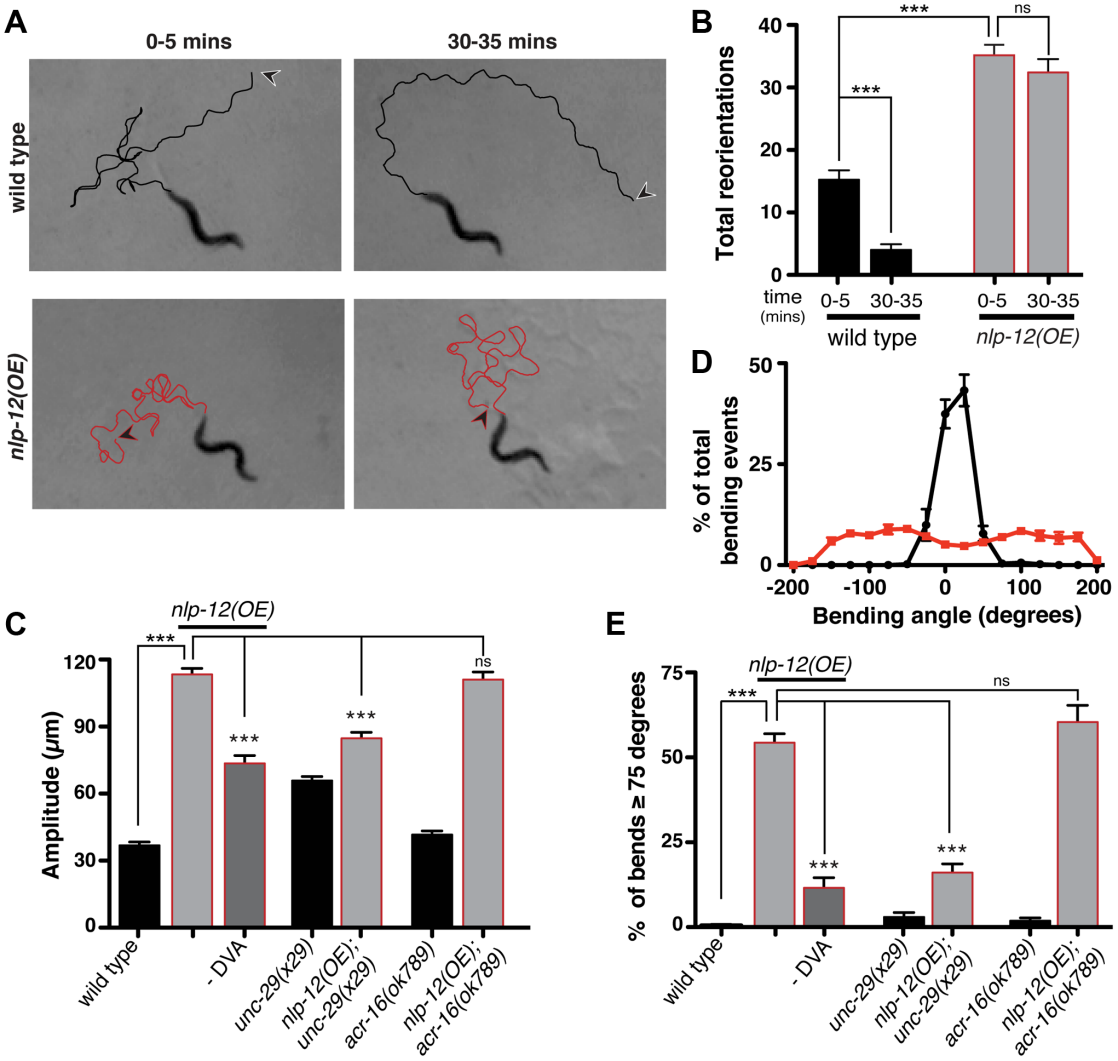
Elevated NLP-12 signaling induces a chronic local search-like state. (**A**) Representative tracks (45 s) of wild type (black lines) and *nlp-12(OE)* (red lines) animals during 0–5 and 30–35 minute intervals after removal from food as indicated. *nlp-12(OE)* refers to a transgenic strain (*ufIs104*) stably expressing high levels of the wild type *nlp-12* genomic sequence. Note that high angled reorientations still persist during the 30–35 minute interval for *nlp-12(OE)* in contrast to the long forward run of the wild type animal. The starting point of each track is indicated by an arrowhead (wild type: black; *nlp-12(OE)*: red). (**B**) Total directional reorientations measured during 0–5 and 30–35 minute intervals following removal from food for wild type (n = 8) and *nlp-12(OE)* (n = 15) animals. (**C**) Average body bend amplitude for the genotypes indicated. –DVA refers to DVA ablation (WT: n = 11, *nlp-12(OE)*: n = 15, *nlp-12(OE)* -DVA: n = 10, *unc-29*: n = 30, *unc-29;nlp-12(OE)*: n = 22, *acr-16*: n = 16, *acr-16;nlp-12(OE)*: n = 15). (**D**) Frequency distribution of bending angles for wild type (black) and *nlp-12(OE)* (red) animals monitored on bacteria seeded plates for 30 s (WT: n = 12, *nlp-12(OE)*: n = 15). (**E**) Bar graph depicting total percentage of bending angles ≥75° for the genotypes indicated (WT: n = 12, *nlp-12(OE)*: n = 14, *nlp-12(OE)* -DVA: n = 9, *unc-29*: n = 10, *unc-29;nlp-12(OE)*: n = 12, *acr-16*: n = 10, *acr-16;nlp-12(OE)*: n = 12). Note that wild type worms rarely execute bends ≥75°. Bars represent mean (±SEM). For (B), (C) and (E) ***, p<0.0001 by ANOVA with Sidak's post-hoc test.

Our finding that NLP-12 signaling was required for animals to appropriately modulate body bends and turning during foraging suggested that altering endogenous NLP-12 levels would be sufficient to modify motor output. To test this idea, we expressed the *nlp-12* genomic sequence and native promoter at high copy levels in wild type animals. We found that the locomotory pattern was dramatically altered in transgenic animals overexpressing a stably integrated *nlp-12* transgene (*nlp-12*(OE); *ufIs104*). To investigate effects of *nlp-12* overexpression on foraging, we examined local search responses to food deprivation. *nlp-12* overexpression increased high-angle turning in the initial five minutes following removing from food by more than 2-fold, and induced occasional bouts of coiling (Fig. 5A, B). Notably, the effects of *nlp-12* overexpression were not significantly attenuated by prolonged food deprivation and persisted even in the presence of food. Our overexpression analysis suggests that genetically elevating levels of NLP-12 signaling compromised modulatory control of behavioral responses to changes in food availability, producing a chronic local search-like behavioral state.

To investigate the effects of *nlp-12* overexpression in more detail, we also examined body bend amplitude and bending angles for both the wild type and *nlp-12*(OE) strain (Fig. 5C–E) during exploratory movement in the presence of food. Body bend amplitude was increased by roughly 3-fold in *nlp-12*(OE) animals (Fig. 5C). We quantified bending angles as deviations from the midline using the midpoint of the body as reference. For wild type animals, body bend angles rarely exceeded 75°. In contrast, bending angles in the *nlp-12*(OE) strain were more broadly distributed, often exceeding 150° (Fig. 5D, E). These effects of *nlp-12* overexpression were significantly attenuated after DVA ablation, yet were not reversed completely. Although we confirmed that the DVA cell body was eliminated in our ablations, we noted that residual mCherry signal associated with the DVA process often remained, suggesting continuing secretion of overexpressed NLP-12 from the process may account for the partial effects of DVA ablation. Alternatively, the partial effects of DVA ablation may indicate that the *nlp-12* promoter drives very low levels of expression from other cells that were not detectable with our reporter. Taken together, our findings indicate that increasing NLP-12 levels is sufficient to alter locomotor activity by enhancing the depth of sinusoidal body bends and increasing bending angles.

As our previous analysis indicated NLP-12 signaling was important for L-AChR*(gf)* effects on movement, we next asked whether normal L-AChR function was required for the effects of *nlp-12* overexpression. UNC-29 is an essential L-AChR subunit and mutation of *unc-29* leads to a complete absence of L-AChR mediated currents from body wall muscles. Loss of L-AChR function causes slowed movement, but does not disrupt the sinusoidal locomotory pattern [17], [22], [53]. The increases in body bend amplitude and bending angles that occurred with *nlp-12* overexpression were significantly reduced by mutation of *unc-29* (Fig. 5C, E). These results suggest NLP-12 mediated effects on the motor pattern require intact L-AChR signaling at the NMJ. In contrast, deletion of *acr-16*, an essential subunit of a second population of iAChRs that mediate much of the evoked current at the NMJ [17], did not alter bending angles or body bend amplitude in *nlp-12*(OE) animals. Thus, there is a specific requirement for L-AChR, but not N-AChR, function in NLP-12 enhancement of body bend amplitude and turning behavior. While the precise synaptic organization of L-AChRs and ACR-16 receptors at the neuromuscular junction has not been determined, our findings suggest the effects of NLP-12 are mediated through synapses where L-AChR signaling predominates.

### The time course of L-AChR synaptic currents is regulated by NLP-12/CCK

**Figure 6:**
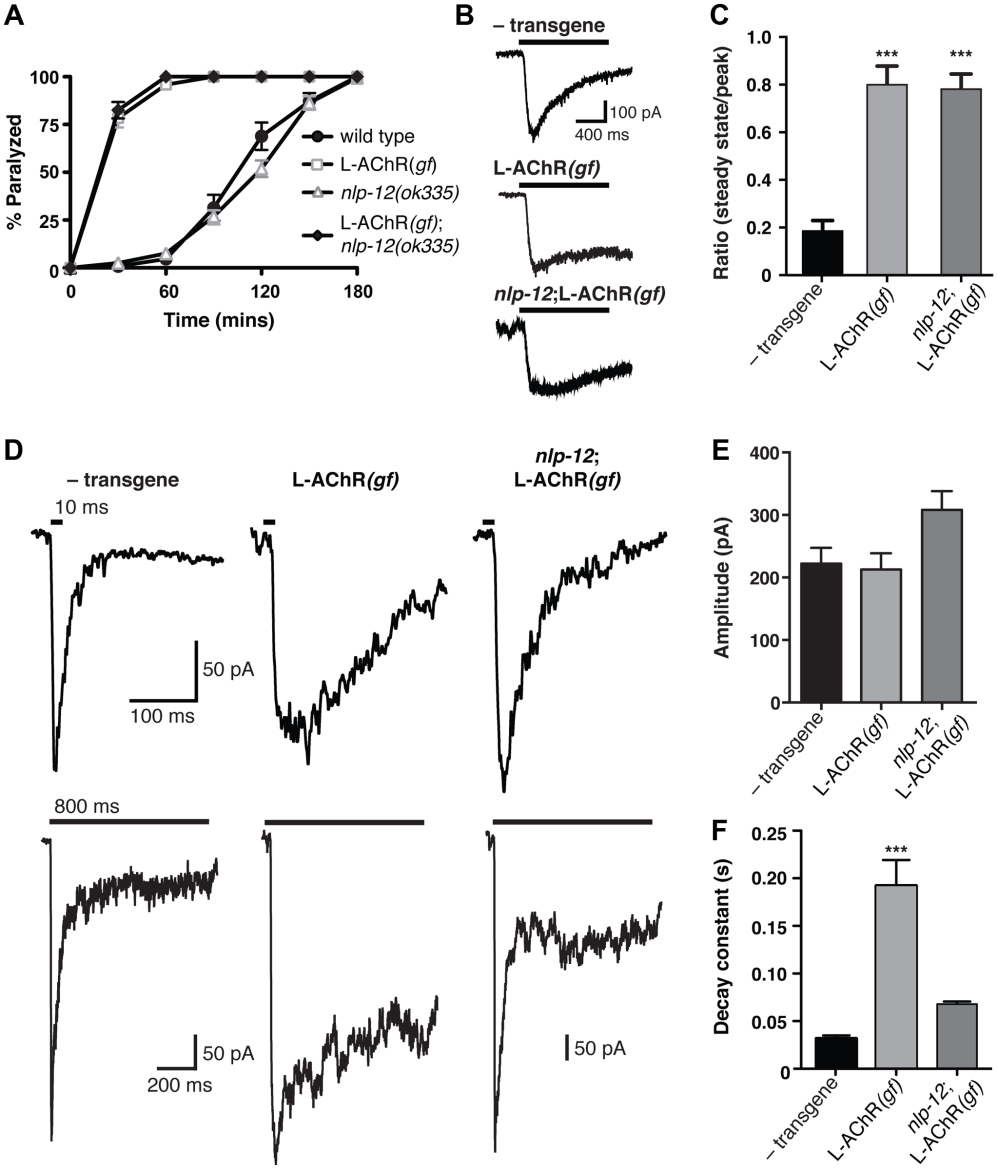
Evoked synaptic responses are prolonged in L-AChR(*gf*) animals and require *nlp-12*. (**A**) Time course of paralysis in the presence of the L-AChR agonist levamisole (200 µM) for wild type, L-AChR*(gf)*, *nlp-12* mutants and *nlp-12*;L-AChR*(gf)* animals as indicated. Each data point represents the mean (± SEM) for at least 10 trials. (**B**) Current responses to pressure application (1 s) of levamisole (500 µM) recorded from body wall muscles of control (− transgene) (n = 5), L-AChR*(gf)* (n = 6) and *nlp-12*;L-AChR(*gf*) (n = 4) animals as indicated. Holding potential was −60 mV. (**C**) The ratio of the current amplitude 1 s after the start of levamisole application (steady state) to the peak current for control, L-AChR(*gf*), and *nlp-12*;L-AChR(*gf*) animals as indicated. (**D**) Current responses to photostimulation of motor neurons (10 ms, upper or 800 ms, lower) recorded from body wall muscles of control, L-AChR(*gf*), and *nlp-12*;L-AChR(*gf*) animals as indicated. Holding potential was −80 mV. Recordings were made in the presence of the N-AChR antagonist dHβE (10 µM) to isolate the L-AChR mediated current. (**E, F**) Average amplitude (E) decay time constant (F) for synaptic responses to 10 ms motor neuron photostimulation for the genotypes indicated. The decay phase of L-AChR mediated evoked current responses were fit with a single exponential. Bars represent mean (± SEM) of amplitude and decay time constant values calculated for responses from control (n = 19), L-AChR*(gf)* (n = 11), and *nlp-12*;L-AChR*(gf)* (n = 11) animals. ***, p<0.0001 by ANOVA with Sidak's post-hoc test. Each strain stably expressed the *Pacr-2::ChR2-GFP* transgene (*ufIs23*) for photostimulation of motor neurons.

Neuromodulators can achieve their effects by regulating the release of classical neurotransmitters, altering post-synaptic receptor functional properties, or otherwise altering excitability. We sought to distinguish between these possible models for *nlp-12* action. As our previous analyses showed the importance of L-AChR function in NLP-12 modulation, we first examined the effects of *nlp-12* deletion on post-synaptic L-AChR functional properties. To facilitate our analysis, we returned to the L-AChR(*gf*) strain where a behavioral requirement for *nlp-12* was most apparent. L-AChR(*gf*);*nlp-12* animals paralyzed more rapidly to the specific agonist levamisole than the wild type, resembling transgenic animals that were wild type for *nlp-12* (Fig. 6A). These results suggested L-AChR function was not appreciably altered by mutation of *nlp-12*. To examine this directly, we made whole-cell recordings of current responses to exogenous levamisole from body wall muscles. The time course of levamisole-evoked currents in L-AChR(*gf*) animals was prolonged dramatically compared to the wild type, while peak amplitude was unaffected (Fig. 6B) – a result consistent with the accelerated time course of levamisole-induced paralysis described above. However, mutation of *nlp-12* did not produce obvious changes in the magnitude or duration of levamisole-evoked current responses (Fig. 6B, C). These results demonstrate that the effects of NLP-12 are unlikely to be mediated through direct modulation of post-synaptic L-AChR function.

A previous study found that treatment with the cholinesterase inhibitor aldicarb increased ACh release from motor neurons, and that these effects required *nlp-12* [39]. To investigate whether the effects of NLP-12 we observed in our behavioral experiments may involve a similar mechanism, we recorded endogenous excitatory synaptic events as well as synaptic events evoked by photostimulation of motor neurons. We did not observe a significant change in the rate or amplitude of endogenous excitatory events with expression of the L-AChR(*gf*) transgene or with deletion of *nlp-12* (Fig. S5). In contrast, L-AChR(*gf*) expression produced a dramatic change in evoked synaptic currents. These specific effects on evoked currents may suggest that the lifetime of ACh in the synaptic cleft is a primary factor in determining the duration of endogenous synaptic events, while the duration of evoked events are less strongly impacted by the action of cholinesterase. We measured evoked synaptic currents in response to photostimulation of motor neurons using a transgenic strain (*ufIs23*) stably expressing Channelrhodopsin-2 (ChR2) in cholinergic motor neurons. In wild type animals, excitatory evoked currents reflect synaptic activation of both L-AChRs and N-AChRs. The rapid peak phase of the synaptic current is primarily mediated by N-AChRs, and can be eliminated by deletion of the *acr-16* gene or application of the N-AChR specific antagonist, dihydro-β-erythroidine (dHβE) [17]–[19]. Conversely, the prolonged component of the synaptic current is mediated through L-AChR activation. The peak amplitude of evoked synaptic currents to brief light exposure (10 ms) was not altered appreciably by L-AChR(*gf*) expression; however, the duration of evoked current responses was increased significantly (Fig. S6). To investigate this effect in more detail, we isolated the L-AChR-mediated component of the evoked synaptic current using dHβE. The decay time of the L-AChR synaptic current was increased dramatically by L-AChR(*gf*) expression without a significant change in current amplitude (Fig. 6D–F). The increased duration of evoked synaptic currents became even more apparent with prolonged (800 ms) stimulation of motor neurons (Fig. 6D, lower). Deletion of *nlp-12* significantly reduced the duration of evoked responses in L-AChR(*gf*) animals (65±1% reduction, p<0.001), such that the average decay time of evoked L-AChR currents in *nlp-12* mutants expressing the L-AChR(*gf*) transgene was not statistically different from that of the wild type (Fig. 6D, F). Thus, the duration of post-synaptic currents evoked by ACh release from motor neurons were normalized by deletion of *nlp-12*, whereas current responses to drug application onto muscles were largely unaffected. These results support a model where NLP-12 increases the duration of synaptic currents by enhancing ACh release from cholinergic motor neurons.

## Discussion

Understanding how organisms appropriately adjust behavioral responses to their environment requires knowledge of how sensory information is transformed by neural circuits into motor outputs. Here, we provide a mechanism by which dopaminergic modulation of neuropeptide signaling alters neuromuscular excitability and shapes *C. elegans* foraging behavior. By elevating neuromuscular excitability, we revealed a state-dependent role for the *C. elegans* cholecystokinin (CCK) homolog NLP-12 in modulating motor behavior. Behavioral modulation by NLP-12 was particularly evident during local food searching, a motor program in which body bend amplitude and the frequency of high-angle turning is transiently increased in response to changes in food availability. NLP-12 signaling was regulated by the D1-like dopamine receptor DOP-1, and DOP-1 signaling was also required for normal food searching behavior. Our results show that *C. elegans* dynamically modulate their motor pattern in response to changes in food availability, through dopaminergic regulation of NLP-12/CCK signaling. Remarkably, similar interactions between dopamine and cholecystokinin occur in the mammalian brain [54], [55], suggesting this may be a conserved mechanism for generating context-dependent behavioral responses.

Behavioral deficits associated with loss of specific neuromodulators are often subtle and transient, owing to the fact that neuromodulatory effects are typically delimited to specific patterns of behavior. As a consequence of this, efforts to identify molecular components of specific neuromodulatory pathways using forward genetic approaches have met with only limited success. We developed a genetic strategy to overcome these hurdles. We engineered a mutation into the pore-forming domains of muscle L-AChR subunits to facilitate studies of neuromodulation in shaping *C. elegans* movement. Incorporation of mutant L-AChR subunits heightened sensitivity to pharmacological agents that increased post-synaptic iAChR activation, and prolonged the duration of evoked synaptic currents in electrophysiology studies. Using this approach to increase muscle synapse activity produced exaggerated sinusoidal movement, a behavioral phenotype distinct from previously reported mutations that enhanced muscle excitability (*e.g.* by elimination of inhibitory signaling onto muscles [56]), suggesting that this manipulation would be informative for identifying novel modulators of excitatory transmission and movement. Consistent with this, a recent study employing a similar gain-of-function approach with the neuronal iAChR subunit ACR-2 identified roles for the *flp-1* and *flp-18* neuropeptides in regulating the activity of motor neurons [57].

The locomotory effects we observed with L-AChR(*gf*) expression were dependent upon neuropeptide signaling, as they were reversed by mutation of either *pkc-1* nPKCε/η, *egl-3* PC2 or *egl-21* Carboxypeptidase E. Disruption of *egl-3* leads to loss of the vast majority of mature *nlp* and *flp* neuropeptides in *C. elegans*, including NLP-12, while mutation of either *pkc-1* or *egl-21* affects a smaller subset of neuropeptides [29], [30], [35]. The most potent neuropeptide modifier of L-AChR(*gf*) locomotory behavior in our assays was NLP-12, and based on our analysis, we would predict that NLP-12 signaling is directly or indirectly dependent on normal PKC-1 and EGL-21 function. NLP-12 overexpression altered movement and produced increases in body bend amplitude that were qualitatively similar to those observed for L-AChR(*gf*) animals. Moreover, normal L-AChR function was required for the full effects of NLP-12 overexpression. These data suggested that one avenue for NLP-12 effects on the motor pattern was through direct modulation of neuromuscular transmission. In support of this, we found that L-AChR(*gf*)-mediated increases in the duration of synaptic currents evoked by photostimulation of motor neurons were reversed almost completely in *nlp-12* mutants.

Prior work has demonstrated that acute pharmacological treatment with the cholinesterase inhibitor aldicarb potentiates ACh release from motor neurons via a mechanism that requires NLP-12 [39]. As a primary effect of aldicarb treatment is muscle contraction, it was suggested that muscle contraction may trigger activation of the stretch–sensitive DVA neuron and subsequent release of NLP-12, enhancing neuromuscular signaling. Thus, under certain conditions muscle contraction may modulate the motor pattern through a proprioceptive-like mechanism. Our genetic, electrophysiological and behavioral data support and extend this model. Expression of L-AChR(*gf*) causes chronic muscle contraction in a manner similar to aldicarb treatment, and both manipulations heighten activation of synaptic L-AChRs. We found that both NLP-12 and DVA are required for the behavioral effects of L-AChR(*gf*) expression, and *nlp-12* deletion reduces the duration of L-AChR mediated synaptic currents. These results are consistent with the model that NLP-12 release from DVA acts in a feedforward pathway required for the behavioral phenotype of L-AChR(*gf*) animals.

Our analysis also revealed novel roles for DVA and NLP-12 signaling in shaping alternative motor responses during local food searching. Neuromodulatory signaling allows anatomically fixed circuits to have flexibility in their outputs. Owing to detailed electron microscopy and laser ablation studies, we have long known the identity and connectivity patterns of the principal neurons involved in the control of *C. elegans* movement [13], [15], [45], [58], [59]. Nonetheless, defining neuromodulatory effects on motor circuit activity and behavior has remained a challenge. Our findings provide support for a model where sensory information about food availability is translated into context-dependent motor output by dopaminergic regulation of NLP-12 release from DVA and NLP-12 modulation of locomotory output. We propose that the DVA neuron integrates proprioceptive and dopaminergic mechanosensory information. In this model, NLP-12 release reflects contributions from both forms of sensory input. In support of this view, we show that dopamine elicits increases in reorientation in a *dop-1* dependent manner without a requirement for increased muscle synapse activation. Conversely, mutation of *cat-2*/tyrosine hydroxylase does not alter behavioral changes elicited by increased muscle synapse activity. Thus, our findings suggest heightened signaling through either sensory modality (proprioceptive or dopaminergic mechanosensory) is sufficient to elicit altered NLP-12 release and behavioral changes. We envision that the precise nature of the behavioral changes reflect characteristics of the pattern of DVA activation as well as the activity of other neuronal pathways that contribute to each behavior.

In principle, the context-dependent effects of NLP-12 could reflect altered sensitivity to the peptide during particular patterns of motor activity or altered release of the peptide in response to changing sensory information. Our analysis supports the latter possibility. Food availability is monitored through dopaminergic mechanosensory signaling (ADE, PDE, and CEP neurons), as well as through activation of specific classes of olfactory and gustatory neurons (AWA, AWC and ASE) [13], [14], [44]. Neuropeptide signaling plays important roles in the regulation of olfactory neuron responses to food [46], [60]; however, we have had only a limited understanding of how sensory representations of food availability are translated into altered patterns of behavior. A major source of synaptic input to DVA is through the dopaminergic neuron PDE. Prior work showed normal area-restricted search behavior requires dopaminergic signaling and implicated involvement of PDE [14]. Our results support the notion that an initial period of local searching immediately following removal from food is accomplished through DVA activation via dopaminergic inputs from PDE and context-dependent stimulation of NLP-12 release. This pathway may act in parallel with sensory information carried by olfactory neurons in order to reinforce the local searching motor pattern. Acute dopamine treatment rapidly decreased NLP-12 levels in DVA, and these effects were reversed with mutation of the D1-like dopamine receptor *dop-1*. Indeed, acute exposure of *dop-1* mutants to dopamine produced significant NLP-12 accumulation in DVA axons, suggesting additional DOP-1-independent effects of dopamine on DVA. DOP-1 was previously shown to promote ACh release from motor neurons by functioning antagonistically to the D2-like receptor DOP-3 and coupling to Gαq/EGL-30 and phospholipase C (PLCβ/EGL-8) [52], [61]. Our results suggest DOP-1 may similarly act in opposition to another D2-like dopamine receptor to regulate NLP-12 release from DVA during local searching. *dop-3* expression has not been reported in DVA and we did not observe any effect of *dop-3* mutations on local food searching behavior (Figure 4E). A second D2-like dopamine receptor is encoded by the *dop-2* gene, and *dop-2* transcriptional reporters are expressed in a variety of head neurons, as well as some unidentified tail neurons [62]. Thus, *dop-2* may be a good candidate as an additional participant in dopamine regulation of NLP-12 release from DVA. Alternatively, the NLP-12 accumulation in *dop-1* mutants may reflect homeostatic changes due to loss of *dop-1*. The olfactory neurons involved in food-sensing promote local searching behavior through synaptic contacts onto interneurons involved in the control of movement [13], [46], [60]. DVA has synaptic outputs onto interneurons that are postsynaptic partners of these neurons, in addition to motor neurons. This pattern of connectivity suggests that the modulatory effects of DVA and perhaps NLP-12 may extend beyond motor neurons, allowing for integrated regulation of neuronal activity at several layers of the circuit.

The ligand and GPCR families underlying neuromodulatory signaling are highly conserved throughout the animal kingdom [42],[63],[64]. In particular, NLP-12 shares sequence similarity with mammalian gastrin and cholecystokinin peptides [64]. In mammals, the effects of CCK are mediated through two GPCRs, CCK1R and CCK2R [65]. CKR-2 is a *C. elegans* homologue of mammalian CCK/gastrin receptors and, to date, is the only GPCR that has been shown to bind NLP-12 [37]. We show that CKR-2 is not required for normal local food searching behavior. This result raises the possibility that, as is the case for mammals, NLP-12/CCK effects are not mediated through a single GPCR. There are several genes encoding GPCRs with significant homology to *ckr-2* in the worm genome (*e.g. ckr-1*, *npr-2*, *npr-5*). Of these, *ckr-1* and *npr-2* remain uncharacterized and are good candidates as additional components in NLP-12 regulation of foraging.

Mammalian CCK signaling regulates food intake by stimulating smooth muscle contractions, intestinal motility and secretion of digestive enzymes, as well as acting centrally to promote satiety [66]. Recent work in *C. elegans* has suggested NLP-12 plays similar roles in promoting digestive enzyme secretion and regulating fat storage [37]. Our work provides evidence that dopaminergic regulation of NLP-12 release and motor circuit activity is central to local food searching behavior. We suggest these dual roles for the conserved NLP-12/CCK signaling pathway in *C. elegans* enable a strategy for optimizing resource utilization through coordinated regulation of metabolic processes and food searching behavior.

## Materials and Methods

### Strains

All nematode strains were maintained on agar nematode growth media seeded with OP50 at room temperature (22–24°C) as described previously [67]. The wild type reference animals for all cases are the N2 Bristol strain. The following strains were used or generated in this work: IZ818: *ufIs47 [Pmyo-3:: unc-38, Pmyo-3::unc-29, Pmyo-3:: lev-1]*, IZ236: *ufIs6 [Pmyo-3:: unc-38(V/S), Pmyo-3::unc-29(L/S), Pmyo-3:: lev-1(L/S)]*, VC731: *unc-63 (ok1075)I*, IZ1260: *unc-63(ok1075);ufIs6*, IZ801:*ufIs23[Pacr-2::ChR2-GFP]*; IZ806: *ufIs23;ufIs6*, RB781: *pkc-1(ok563)V*, KP2342: *pkc-1(nu488)V*, IZ1001: *pkc-1(ok563);ufIs6*, VC671: *egl-3(ok979)V*, IZ1006: *egl-3(ok979);ufIs6*, KP2018: *egl-21(n476)IV*, IZ825: *egl-21(n476);ufIs6*, IZ908: *nlp-12(ok335)I*, IZ681: *nlp-12(ok335);ufIs6*, IZ1304: *nlp-12(ok335);ufEx430[Pnlp-12::nlp-12::SL2::mCherry]*, IZ1099: *ufEx364[Pnlp-12::nlp-12::SL2::mCherry]*, IZ1099: *ufIs6; ufEx364[Pnlp-12::nlp-12::SL2::mCherry]*, IZ1310: *ufEx432[Pnlp-12::mCherry]*, IZ1432: *ufEx475[Pnlp-12::Tetanus]*, IZ1480: *ufIs6; ufEx475[Pnlp-12::Tetanus]*, IZ1152: *ufIs104[Pnlp-12::nlp-12 genomic locus]*, IZ1313: *ufIs104;ufEx433[Pnlp-12::mCherry]*, LSC0032: *ckr-2(tm3082)III*, IZ74: *unc-29(x29)I*, IZ1729: *unc-29(x29);ufIs104*, IZ57: *acr-16(ok789)V*, IZ1728: *acr-16(ok789);ufIs104*, LX645: *dop-1(vs100)X*, IZ1512: *dop-1(vs100)X;ufEx498 (Pnlp-12::dop-1)*, LX636: *dop-1(vs101)*X, LX703: *dop-3(vs106)X*, IZ1299: *lin-15(n765ts)X;ufEx428[Pnlp-12::nlp-12::Venus-YFP*], XP2: *dop-1(vs100) lin-15(n765ts) X*, IZ1479: *dop-1(vs100) lin-15(n765ts);ufEx428[Pnlp-12::nlp-12::Venus-YFP*], IZ1513: *dop-1(vs100) lin-15(n765ts);ufEx428[Pnlp-12::nlp-12::Venus-YFP*]*;ufEx499 [Pnlp-12::dop-1]*, LX831: *vsIs28[Pdop-1::GFP];vsIs33[Pdop-3::RFP]*, IZ1477: *vsIs28[Pdop-1::GFP]; ufEx440[Pnlp-12::nlp-12::SL2::mCherry]*, IZ1605: *cat-2(n4547) ufIs6 II*.

### Molecular biology

#### L-AChR subunit constructs

Wild type *unc-38*, *unc-29* and *lev-1* cDNAs were PCR amplified using primers designed to the start and stop sites of each gene and subcloned into the NheI/SacI site of a plasmid containing the *myo-3* promoter to generate pLJ2 [*Pmyo3::unc-38*], pLJ3 [*Pmyo3::unc-29*] and pLJ6 [*Pmyo3::lev-1*]. L-AChR(*gf*) constructs (pMF1 [*Pmyo3::unc-38(V/S)*], pLJ8 [*Pmyo3::unc-29(L/S)*] and pLJ9 [*Pmyo3::lev-1(L/S)*]) were generated by PCR-based site directed mutagenesis using mutant primers with the respective wild type cDNA constructs as templates.

#### Other constructs

An *nlp-12* genomic fragment (−320 bp to +379 bp relative to the translational start site) or *nlp-12* promoter fragment (−320 bp relative to start) was cloned into pENTR-D-TOPO to generate Gateway entry vectors. The entry vectors were recombined with the Gateway destination vectors pDest-27 (SL2::mCherry), pDest-17 (mCherry), pDest-25 (VenusYFP), pDest-34 (Tetanus toxin light chain) or pDest-60 (*dop-1*) to generate the expression plasmids pCL19 [*Pnlp-12::NLP-12::SL2::mCherry*], pCL27 [*Pnlp-12::mCherry*], pCL12 [*Pnlp-12::NLP-12-VenusYFP*], pCL8 [P*nlp-12::Tetx*] and pCL41 [*Pnlp-12::dop-1*] respectively. All plasmids were confirmed by sequencing (Genewiz). Detailed cloning information for generation of the destination vectors is included in the supporting information (Text S1).

### Transgenics

Transgenic strains were obtained by microinjection of one or more of the following plasmid DNAs (10–30 ng/ul) into the germ line: pLJ2 [*Pmyo3::unc-38*], pLJ3 [*Pmyo3::unc-29*], pLJ6 [*Pmyo3::lev-1*], pMF1[*Pmyo3::unc-38 (V/S)*], pLJ8[*Pmyo3::unc-29 (L/S)*], pLJ9 [*Pmyo3::lev-1 (L/S)*], pCL8 [P*nlp-12::Tetx*], pCL12 [*Pnlp-12::NLP-12-VenusYFP*], pCL19 [*Pnlp-12::NLP-12::SL2::mCherry*], pCL27 [*Pnlp-12::mCherry*] and pCL41 [P*nlp-12::*Dop-1]. The *nlp-12(OE)* strain (*ufIs104*) was generated by integration of a 1.76 kb PCR product containing the *nlp-12* promoter and genomic locus (−354 bp to +1407 bp relative to the transcriptional start). Germline transformation was monitored either by coinjection with the *lin-15* rescuing plasmid pL15EK, or by coinjection with fluorescent reporters expressed in the pharynx (pHP6 [*Plgc-11::GFP*]) or intestine (*Pelt-2::GFP*). Multiple independent extrachromosomal lines were obtained for each transgenic strain and data presented are from a single representative transgenic line unless noted otherwise. Stably integrated lines were generated by X-ray integration and outcrossed at least four times to wild type.

### RNAi screen

RNAi feeding experiments were essentially performed as described previously [68]. Briefly, bacteria containing RNAi clone targeting various proneuropeptide genes were cultured in LB media containing 50 ug/ml ampicillin. Bacteria were seeded on NGM agar plates containing 1 mM IPTG together with ampicillin and tetracycline and allowed to dry at room temperature. L4 stage wild type or L-AChR(*gf*) worms were placed on RNAi seeded plates and allowed to reproduce at 20°C. Staged young adult F2 worms were transferred to a fresh plate and visually assessed for altered movement compared to a control strain plated on bacteria expressing the empty vector. Scoring was performed blind and a positive hit was assigned to a plate if at least 25% of animals were scored as showing altered movement. Alterations in movement were scored by visually monitoring track amplitudes during spontaneous movement as well as motor responses to tail touch. Plates were scored in duplicate and movement velocity was quantified for the best candidates by counting body bend frequency in the presence of bacterial food. RNAi targeting the *unc-22* gene was used as a positive control. From this analysis we identified five neuropeptide precursors (*nlp-2*, *nlp-3*, *nlp-7*, *nlp-12*, *nlp-15*) that produced varying degrees of L-AChR(*gf*) suppression when downregulated by RNAi. We confirmed these effects in assays of the corresponding deletion strains in combination with L-AChR(*gf*). Of these, *nlp-12* deletion produced the strongest and most consistent effects.

### Behavioral assays

All behavioral analyses were performed using staged populations of young adult animals (24 h following L4) at room temperature (22–24°C). Strains were scored in parallel, with the researcher blinded to genotype. Movies and still images for behavioral analyses were obtained using an Olympus SZ61 upright microscope equipped with a FireWire camera (Imaging Source) and were acquired at a rate of 30 frames/s.

For locomotion assays on agar, individual worms were transferred to fresh 5 cm NGM agar plates thinly seeded with bacteria and allowed to acclimate for 1–2 minutes prior to filming. In all cases 45 s digital video of individual worms were captured at 0.75× magnification and converted into AVI format prior to analysis using WormLab software (MBF Bioscience). Each movie was analyzed using the backtracking module and mean body bend amplitude was calculated. All parameters were kept constant at manufacturer's recommended settings with the exception that thresholding was varied from movie to movie in order to maximize optimal recognition of worms. Track lengths and body lengths were calculated using ImageJ. For analysis of body lengths, the ventral midline was measured from synchronized young adult animals.

Analyses of area-restricted food searching were performed following the protocol outlined in [14] with minor variations. Staged animals were transferred from food plates onto 11 cm NGM agar observation plates without food after removal of excess bacteria. Movement was recorded for a 5-minute period immediately following transfer to the observation plate and again after 30 min following transfer. Movement was analyzed using WormLab software to calculate the average body bend amplitudes and the paths of individual worms were reconstructed from the track baselines. To quantitate changes in direction during food search, reorientations were visually identified and counted manually from digital movies. Omega turns were defined as turns in which the head made contact with the tail, and typically followed long reversals of 3 or more head swings. Reorientations in the absence of omega turns were scored as directional changes with an angle change of >50° within a single body bend. In most cases these trajectory changes either followed a brief cessation of forward movement or a short reversal; however, they also occurred less frequently during forward runs. Dopamine effects on directional reorientation were performed using similar criteria, except that animals were monitored for 5 mins immediately after transfer to salt free plates, with or without 2.5 mM dopamine. Dopamine (salt-free) plates were prepared following the protocol outlined in [48]. Reorientation data for each worm was averaged over a 5 min observation period. Additional behavioral analyses of *nlp-12*(OE) animals were conducted on bacteria seeded plates. Movies were analyzed with WormLab software using the Bending Angle – Single parameter to perform a frame by frame measurement of body curvature during movement. Bending angle is defined as the angle at the midpoint of the worm with respect to the head and tail centroids.

For levamisole assays, staged populations of adult animals (≥10) were transferred to NGM plates containing levamisole (Sigma) at the concentration indicated and assayed for paralysis every 15 min for 2–3 hrs. Worms were scored as paralyzed when they failed to execute at least one body bend after prodding with a pick. In all cases, worms that were unresponsive to mechanical prodding were also paralyzed in the absence of mechanical stimulation.

Movement assays in liquid were carried out essentially as described previously [69]. Briefly, staged single adult animal were transferred without food onto a PCR cap containing 20 µl of M9 buffer. After an equilibration period of 1 min, the number of body bends was counted manually over a 3 min period using a Leica MZ 6 series microscope at 1.5× magnification.

For all pairwise comparisons, statistical significance was determined by two-tailed Student's t-test using GraphPad Prism software. For all multiple comparisons, statistical significance was determined by ANOVA followed by Sidak's test.

### DVA ablation

Laser ablations were conducted using standard methods with a pulsed-UV laser (Photonic Instruments, model: 337-USAS) [70]. Briefly, animals were mounted on agar pads and anesthetized in 5 µl of 20 mM sodium azide. Both mock-ablated and laser-operated animals were processed in parallel. The DVA neuron was identified in transgenic L2/L3 larval animals expressing mCherry under the *nlp-12* promoter. Ablation of DVA was confirmed by an absence of fluorescence in the cell body position in adult animals. Behavioral assays were conducted on young adult animals following a recovery period of roughly two days after ablation.

### Microscopy

Epifluorescent imaging was performed using a Zeiss Axioimager M1 microscope and Axiovision software (Zeiss). Confocal microscopy was performed using a Zeiss Axioskop 2 microscope system and LSM Pascal 5 imaging software (Zeiss). Worms were mounted on agarose pads and immobilized with sodium azide. All images were obtained from staged young adult animals (24 h after the L4 stage) and processed using ImageJ software (open source). For quantitative imaging of NLP-12-VenusYFP fluorescence in DVA axon, animals were orientated with their ventral side facing the objective and imaged immediately anterior to the vulva. Imaging was performed after transfer to salt-free plates containing either a thin lawn of bacteria or 10 mM dopamine for a period of ten minutes. Images were acquired as 0.5 µM confocal slices and maximum intensity projections were utilized for further fluorescence analysis. Raw YFP fluorescence values were normalized to the absolute mean fluorescence of ∼0.175 µm diameter green fluorescent microsphere beads (Molecular Probes).

### Electrophysiology

Postsynaptic currents were recorded from body wall muscles using whole-cell patch clamp electrophysiology as previously described [71], [72]. The extracellular solution typically consisted of 150 mM NaCl, 5 mM KCl, 15 mM HEPES, and 10 mM glucose (pH 7.4, osmolarity adjusted with 20 mM sucrose) with 4 mM MgCl_2_ and 1 mM CaCl_2_. Photostimulation experiments were performed using the same extracellular solution but containing 1 mM MgCl_2_ and 5 mM CaCl_2_. The intracellular fluid (ICF) consisted of 115 mM K-gluconate, 25 mM KCl, 0.1 mM CaCl_2_, 50 mM HEPES, 5 mM Mg-ATP, 0.5 mM Na-GTP, 0.5 mM cGMP, 0.5 mM cAMP, and 1 mM BAPTA (pH 7.4, osmolarity adjusted with 10 mM sucrose). At least 60–90 s of continuous data were used in the analysis of endogenous events. For light evoked recordings, strains expressing ChR2 were illuminated with blue light from Exfo X-Cite series 120 light source, filtered through a GFP excitation filter (450–490 nm). The duration of illumination was defined using a computer-controlled shutter (SmartShutter, Sutter Instrument) and HEKA Patchmaster software. For drug-evoked responses, drugs were pressure ejected using a Picospritzer II (General Valve Corporation). For all recordings, series resistance was compensated 50% and only recordings in which the series resistance was stable throughout the course of the recording were included. Data analysis was performed using Igor Pro (WaveMetrics, Inc.) and Mini Analysis (Synaptosoft, Inc.). Statistical comparisons were made by ANOVA with Sidak's post-hoc test or student's t-test using GraphPad Prism.

## Supporting Information

Figure S1 L-AChR(*gf*) expression at the NMJ. (**A**) Amino acid alignment of the M2 domain for the different iAChR subunits as indicated. Subunits of the pentameric *C. elegans* L-AChR are denoted by the grey box. Mouse brain iAChR subunits are highlighted in green. Red outline denotes the highly conserved leucine residue that was targeted for mutagenesis. 3 of the 5 L-AChR subunits mutated to carry the L/S substitution in L-AChR(*gf*) are further highlighted in yellow. (**B, C**) Confocal images of wild type and L-AChR*(gf)* adult worms expressing either *him-4:*:membrane bound YFP to visualize the outer muscle rows (B) or *acr-2::*mCherry-RAB-3 (C) to visualize synaptic vesicle clusters. Both muscle morphology and the distribution of mCherry-RAB-3 appeared grossly normal, indicating L-AChR(*gf*) expression does not produce muscle toxicity or disruption of neuromuscular synapse development.
(PDF)

Figure S2 L-AChR(*gf*) hypersensitive receptors produce distinct behavioral phenotypes. (**A**) Time course of paralysis in the presence of the cholinergic agonist levamisole (200 µM) for wild type and L-AChR*(gf)* animals as indicated. Each data point represents the mean (± SEM) for at least 10 trials. Treatment with levamisole causes paralysis over time due to prolonged muscle contraction [53], [74], [75]. L-AChR(*gf*) expression accelerated the time course of paralysis, indicating L-AChR(*gf*) receptors are properly transported to the cell surface and act to mediate enhanced muscle excitability. (**B**) Percentage of animals moving after 15 mins in the presence of levamisole for the genotypes as indicated. Stable, muscle-specific expression of cDNAs encoding wild-type copies of the respective L-AChR subunits [L-AChR(*wt*)] did not significantly increase sensitivity to levamisole. To test whether the mutated subunits coassembled with endogenously expressed subunits, we evaluated effects of a mutation in the *unc-63* gene. UNC-63 is an essential α-subunit of the pentameric L-AChR, and mutation of *unc-63* produces resistance to paralysis by levamisole [21]. *unc-63* mutants carrying the L-AChR(*gf*) transgene were also resistant, demonstrating that coassembly with the native UNC-63 subunit is required for L-AChR(*gf*) effects. (**C, D**) Average track length (C) and body length (D) for wild type, L-AChR*(gf)* and L-AChR*(wt)* animals as indicated. Bars represent the mean (±SEM) of values calculated from at least 15 animals. ***, p<0.0001 by ANOVA with Sidak's post-hoc test.
(PDF)

Figure S3 Locomotory phenotypes associated with L-AChR*(gf)* expression require neuropeptide signaling. (**A, B**) Movement trajectories (A) and average track lengths (B) of *egl-3(ok979) and egl-21(n476)* animals. Each black line shows the trajectory of one animal monitored for 45 s on food (n = 5 for both). Values for wild type are taken from D for comparison. *egl-3* and *egl-21* mutants showed pronounced locomotory defects on solid agar even in the absence of the L-AChR*(gf)* transgene. However, swimming behavior was less severely affected (Fig. 2A). The *egl-21(n476)* allele corresponds to an out of frame deletion of 123 bp and is predicted to encode a truncated protein of 132 amino acids. The *egl-3(ok979)* allele corresponds to a 1578 bp deletion, eliminating most of the catalytic domain. (**C, D**) Average body bend amplitude (C) and track length (D) for wild type, L-AChR*(gf), pkc-1* mutants and *pkc-1;*L-AChR*(gf)* animals as indicated. Each bar in C and D represents the mean (±SEM) of values calculated from recordings of at least 15 animals. *pkc-1(ok563)* is a deletion mutation that removes 1673 bp of chromosomal DNA including the 5′ UTR and ATG translational start of *pkc-1*B. *pkc-1(nu448)* is a nonsense mutation that results in a premature stop and a truncated protein product lacking the kinase domain. Body bend amplitude and movement velocity were restored to near wild type levels in either *pkc-1(ok563)* mutants or *pkc-1(ok563/nu448)* trans-heterozygotes (not shown) that carried the L-AChR*(gf)* transgene. (**E**) Movement trajectories of wild type, L-AChR*(gf), pkc-1* mutants and *pkc-1*;L-AChR*(gf)* animals as indicated. Each black line shows the trajectory of one animal monitored for 45 s on food. ***, p<0.0001 by ANOVA with Sidak's post-hoc test.
(PDF)

Figure S4 Requirement of DVA and *nlp-12* for L-AChR(*gf*) locomotor effects. (**A, B**) Average body length (A) and track length (B) for the genotypes indicated. DVA ablation or deletion of the neuropeptide gene *nlp-12* normalizes the movement of L-AChR(*gf*) animals (n = 7 for –DVA). (**C**) Average body bends/min measured in liquid for the genotypes indicated. For A–C, each bar represents the mean (±SEM) of values calculated from recordings of at least 15 animals. ***, p<0.0001, **, p<0.001 by ANOVA with Sidak's post-hoc test. (**D**) Average body bend amplitude for non-transgenic or transgenic wild type and L-AChR*(gf)* animals expressing Tetanus toxin in DVA *[DVA::Tetx]*. Bars represent mean (±SEM) for at least 14 animals. ***, p<0.0001 student's t-test.
(PDF)

Figure S5 The frequency of endogenous excitatory post-synaptic currents is not affected by L-AChR(*gf*) expression. (**A**) Endogenous excitatory synaptic events recorded from body wall muscles of wild type and L-AChR(*gf*) animals as indicated. Holding potential was −60 mV. (**B, C**) Average frequency (B) and amplitude (C) of endogenous excitatory synaptic events in wild type (n = 27), L-AChR(*gf*) (n = 19), *nlp-12* mutant (n = 21), and *nlp-12*;L-AChR(*gf*) (n = 22) animals. Each bar represents mean ± SEM.
(PDF)

Figure S6 L-AChR(*gf*) expression prolongs the slow L-AChR mediated component of synaptic currents. (**A**) Representative muscle current responses to photostimulation of excitatory motor neurons recorded from adult control (n = 10) or L-AChR*(gf)* (n = 7) animals. Black bar indicates duration of motor neuron photostimulation (10 ms). Holding potential was −80 mV. (**B, C**) Average amplitude (B) and decay time (C) of photoevoked currents. Currents were fit with two exponentials (τ1 and τ2) to account for slow and fast currents associated with synaptic activation of L-AChR and N-AChR respectively. Both strains stably express the *Pacr-2::ChR2-GFP* transgene (*ufIs23*).
(PDF)

Text S1 Detailed cloning information for vectors.
(PDF)
