# Supplementary material for: A Conserved Dopamine-Cholecystokinin Signaling Pathway Shapes Context–Dependent Caenorhabditis elegans Behavior
Source: PLoS Genet. 2014 Aug 28;10(8):e1004584. doi: 10.1371/journal.pgen.1004584 (PMC4148232; doi:10.1371/journal.pgen.1004584)
Supplement: Figure S1 — L-AChR(gf) expression at the NMJ. (A) Amino acid alignment of the M2 domain for the different iAChR subunits as indicated. Subunits of the pentameric C. elegans L-AChR are denoted by the grey box. Mouse brain iAChR subunits are highlighted in green. Red outline denotes the highly conserved leucine residue that was targeted for mutagenesis. 3 of the 5 L-AChR subunits mutated to carry the L/S substitution in L-AChR(gf) are further highlighted in yellow. (B, C) Confocal images of wild type and L-AChR(gf) adult worms expressing either him-4::membrane bound YFP to visualize the outer muscle rows (B) or acr-2::mCherry-RAB-3 (C) to visualize synaptic vesicle clusters. Both muscle morphology and the distribution of mCherry-RAB-3 appeared grossly normal, indicating L-AChR(gf) expression does not produce muscle toxicity or disruption of neuromuscular synapse development. (PDF) [file pgen.1004584.s001.pdf]

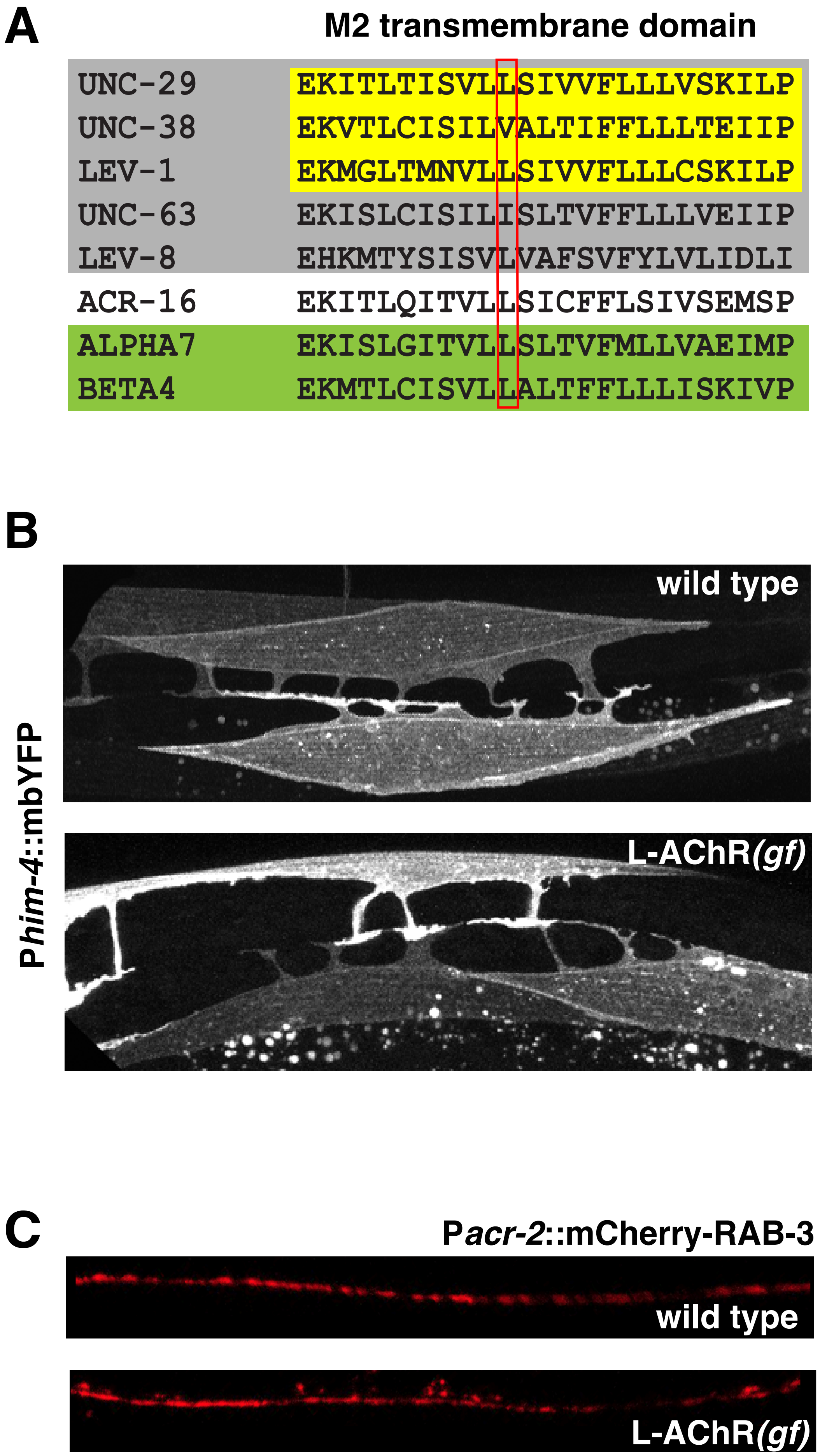
A
M2 transmembrane domain
UNC-29 EKITLTISVLLSIVVFLLLVSKILP
UNC-38 EKVTLCISILVALTIFFLLLTEIIP
LEV-1 EKMGLTMNVLLSIVVFLLLCSKILP
UNC-63 EKISLCISILISLTVFFLLLVEIIP
LEV-8 EHKMTYSISVLVAFSVFYLVLIDLI
ACR-16 EKITLQITVLLSICFFLSIVSEMSP
ALPHA7 EKISLGITVLLSLTVFMLLVAEIMP
BETA4 EKMTLCISVLLALTFFLLLISKIVP
B
Phim-4::mbYFP
wild type
L-AChR(gf)
C
Pacr-2::mCherry-RAB-3
wild type
L-AChR(gf)

Figure S1
