# Supplementary figures and images for: A Conserved Dopamine-Cholecystokinin Signaling Pathway Shapes Context–Dependent Caenorhabditis elegans Behavior
Source: PLoS Genet. 2014 Aug 28;10(8):e1004584. doi: 10.1371/journal.pgen.1004584 (PMC4148232; doi:10.1371/journal.pgen.1004584)

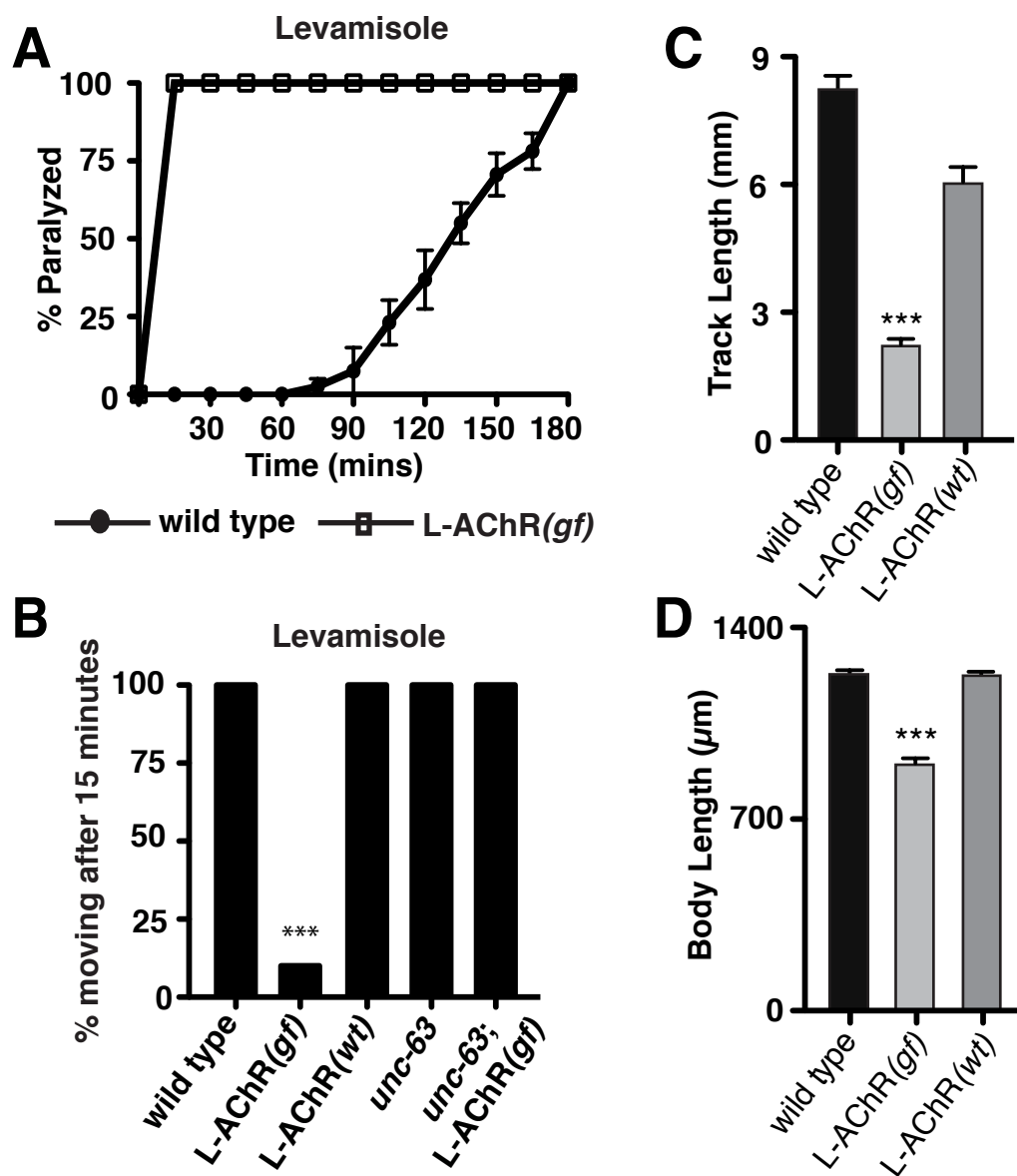

Figure S2

Supplement: Figure S2 — L-AChR(gf) hypersensitive receptors produce distinct behavioral phenotypes. (A) Time course of paralysis in the presence of the cholinergic agonist levamisole (200 µM) for wild type and L-AChR(gf) animals as indicated. Each data point represents the mean (± SEM) for at least 10 trials. Treatment with levamisole causes paralysis over time due to prolonged muscle contraction [53], [74], [75]. L-AChR(gf) expression accelerated the time course of paralysis, indicating L-AChR(gf) receptors are properly transported to the cell surface and act to mediate enhanced muscle excitability. (B) Percentage of animals moving after 15 mins in the presence of levamisole for the genotypes as indicated. Stable, muscle-specific expression of cDNAs encoding wild-type copies of the respective L-AChR subunits [L-AChR(wt)] did not significantly increase sensitivity to levamisole. To test whether the mutated subunits coassembled with endogenously expressed subunits, we evaluated effects of a mutation in the unc-63 gene. UNC-63 is an essential α-subunit of the pentameric L-AChR, and mutation of unc-63 produces resistance to paralysis by levamisole [21]. unc-63 mutants carrying the L-AChR(gf) transgene were also resistant, demonstrating that coassembly with the native UNC-63 subunit is required for L-AChR(gf) effects. (C, D) Average track length (C) and body length (D) for wild type, L-AChR(gf) and L-AChR(wt) animals as indicated. Bars represent the mean (±SEM) of values calculated from at least 15 animals. ***, p<0.0001 by ANOVA with Sidak's post-hoc test. (PDF) [file pgen.1004584.s002.pdf]

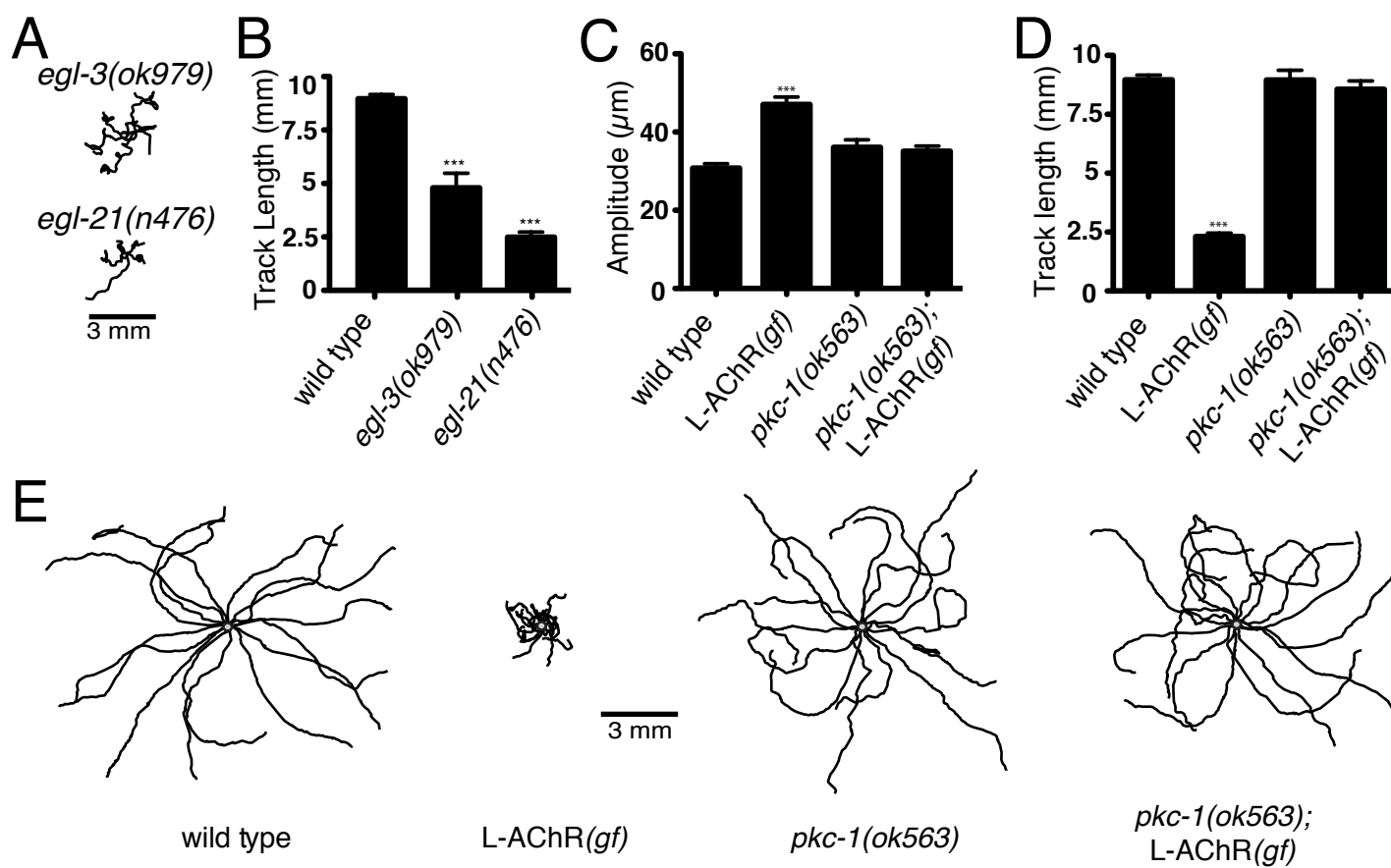

Figure S3

Supplement: Figure S3 — Locomotory phenotypes associated with L-AChR(gf) expression require neuropeptide signaling. (A, B) Movement trajectories (A) and average track lengths (B) of egl-3(ok979) and egl-21(n476) animals. Each black line shows the trajectory of one animal monitored for 45 s on food (n = 5 for both). Values for wild type are taken from D for comparison. egl-3 and egl-21 mutants showed pronounced locomotory defects on solid agar even in the absence of the L-AChR(gf) transgene. However, swimming behavior was less severely affected (Fig. 2A). The egl-21(n476) allele corresponds to an out of frame deletion of 123 bp and is predicted to encode a truncated protein of 132 amino acids. The egl-3(ok979) allele corresponds to a 1578 bp deletion, eliminating most of the catalytic domain. (C, D) Average body bend amplitude (C) and track length (D) for wild type, L-AChR(gf), pkc-1 mutants and pkc-1;L-AChR(gf) animals as indicated. Each bar in C and D represents the mean (±SEM) of values calculated from recordings of at least 15 animals. pkc-1(ok563) is a deletion mutation that removes 1673 bp of chromosomal DNA including the 5′ UTR and ATG translational start of pkc-1B. pkc-1(nu448) is a nonsense mutation that results in a premature stop and a truncated protein product lacking the kinase domain. Body bend amplitude and movement velocity were restored to near wild type levels in either pkc-1(ok563) mutants or pkc-1(ok563/nu448) trans-heterozygotes (not shown) that carried the L-AChR(gf) transgene. (E) Movement trajectories of wild type, L-AChR(gf), pkc-1 mutants and pkc-1;L-AChR(gf) animals as indicated. Each black line shows the trajectory of one animal monitored for 45 s on food. ***, p<0.0001 by ANOVA with Sidak's post-hoc test. (PDF) [file pgen.1004584.s003.pdf]

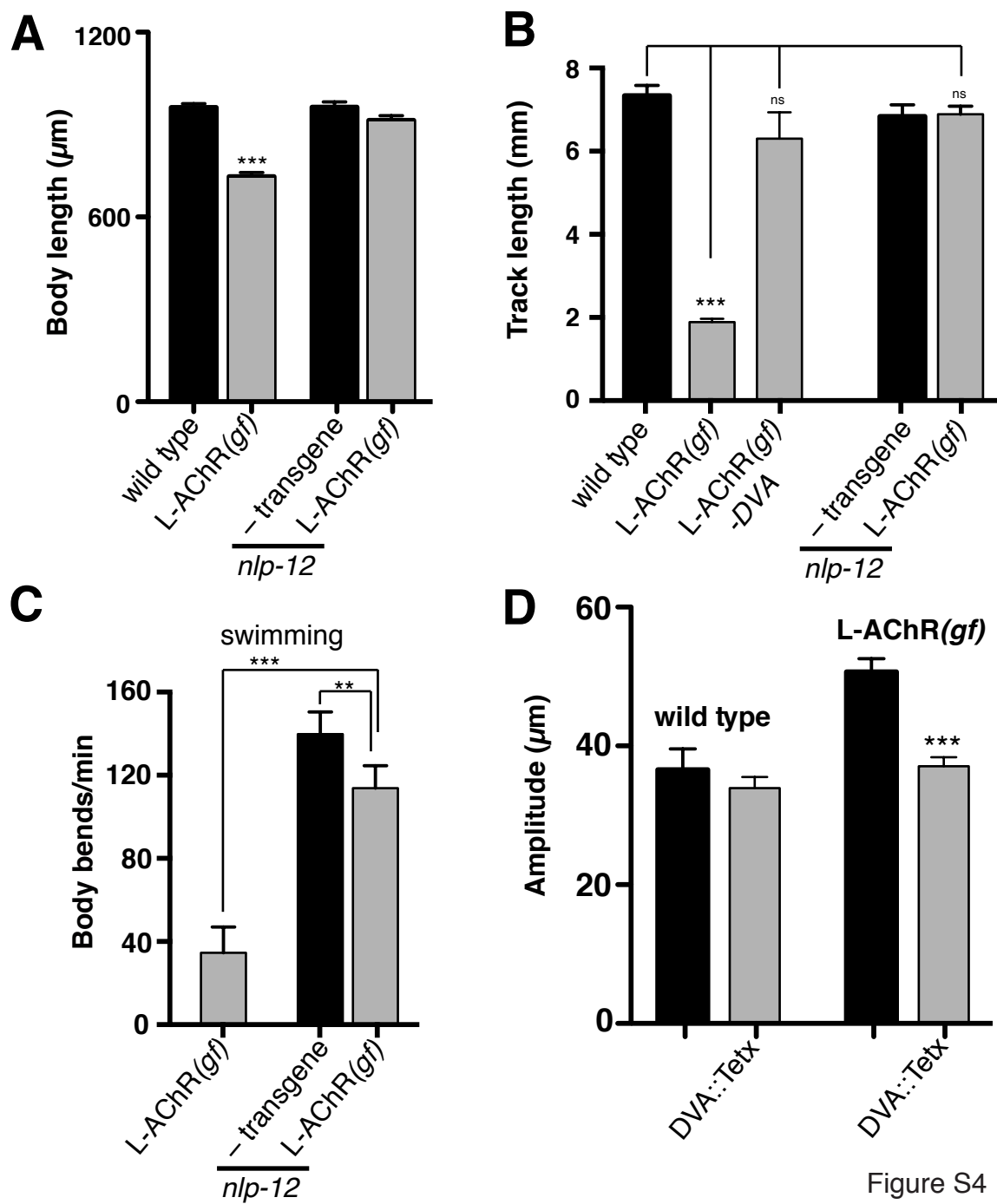

Figure S4

Supplement: Figure S4 — Requirement of DVA and nlp-12 for L-AChR(gf) locomotor effects. (A, B) Average body length (A) and track length (B) for the genotypes indicated. DVA ablation or deletion of the neuropeptide gene nlp-12 normalizes the movement of L-AChR(gf) animals (n = 7 for –DVA). (C) Average body bends/min measured in liquid for the genotypes indicated. For A–C, each bar represents the mean (±SEM) of values calculated from recordings of at least 15 animals. ***, p<0.0001, **, p<0.001 by ANOVA with Sidak's post-hoc test. (D) Average body bend amplitude for non-transgenic or transgenic wild type and L-AChR(gf) animals expressing Tetanus toxin in DVA [DVA::Tetx]. Bars represent mean (±SEM) for at least 14 animals. ***, p<0.0001 student's t-test. (PDF) [file pgen.1004584.s004.pdf]

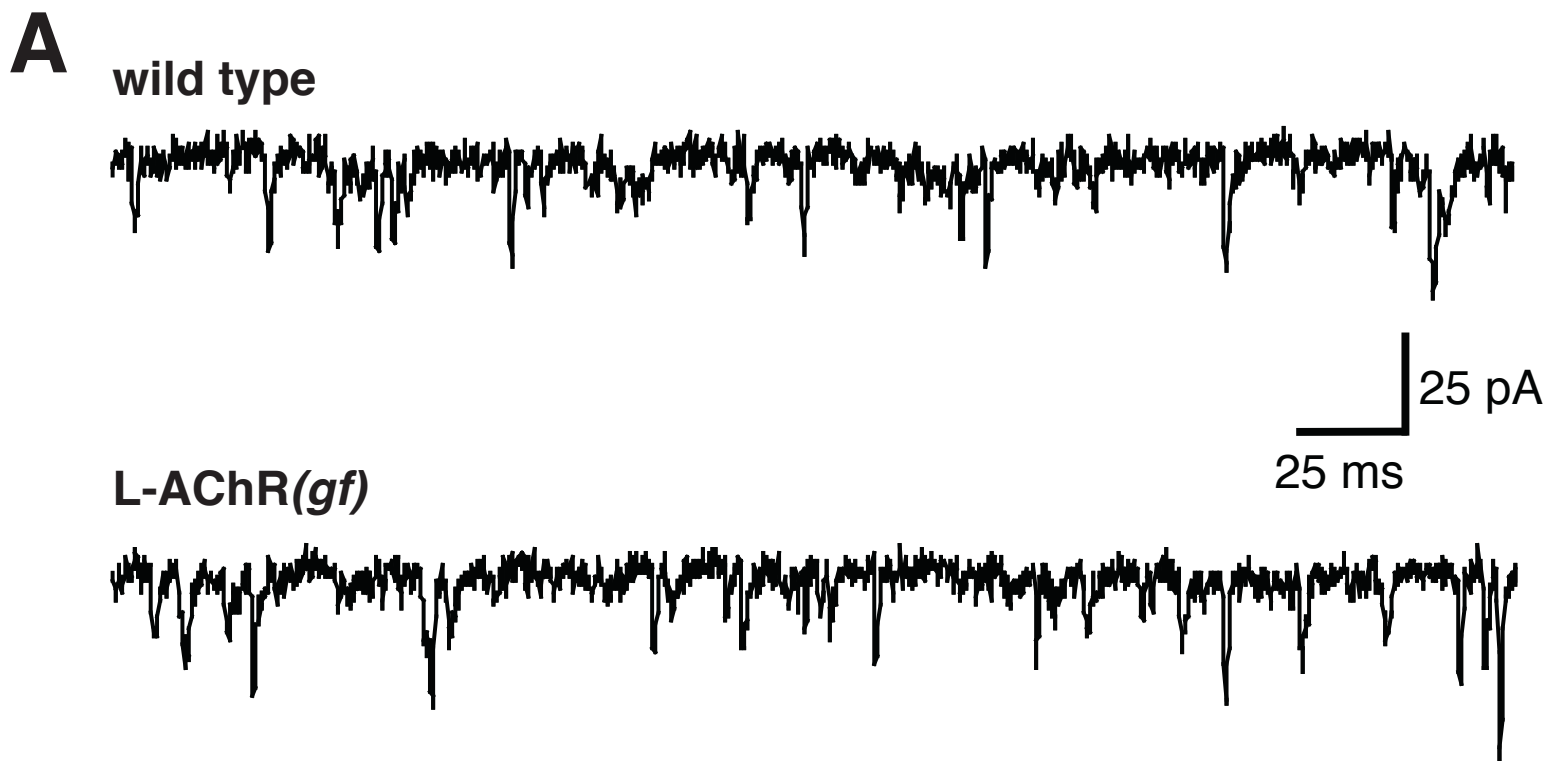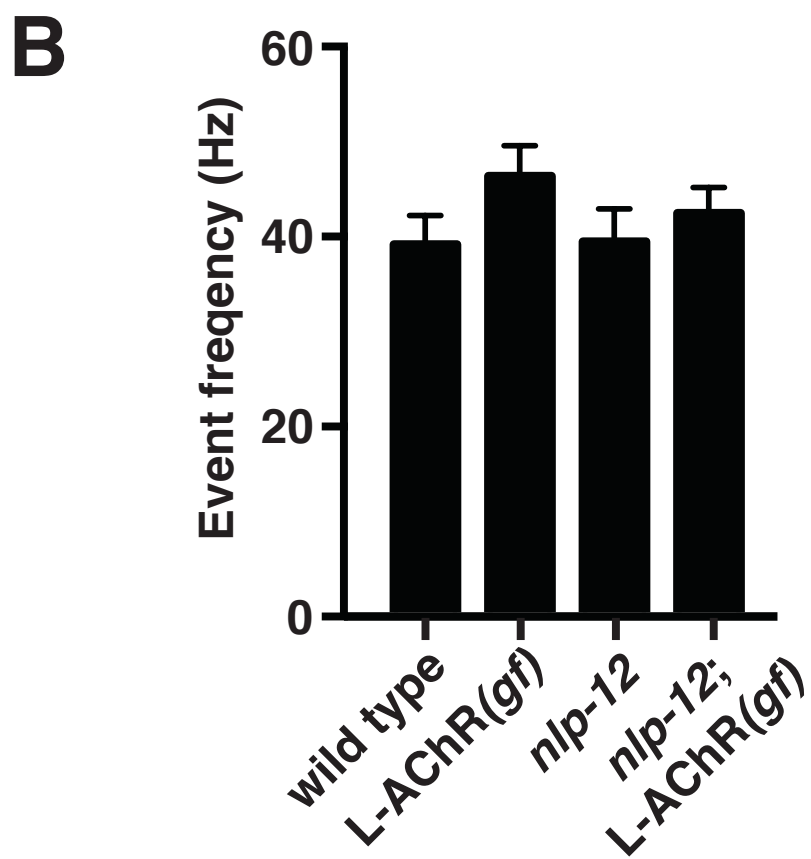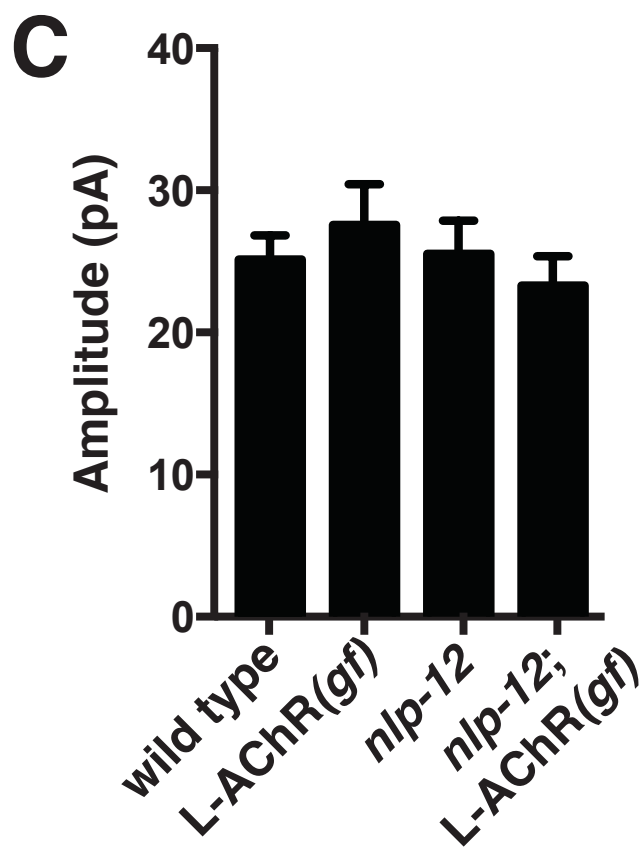

Figure S5

Supplement: Figure S5 — The frequency of endogenous excitatory post-synaptic currents is not affected by L-AChR(gf) expression. (A) Endogenous excitatory synaptic events recorded from body wall muscles of wild type and L-AChR(gf) animals as indicated. Holding potential was −60 mV. (B, C) Average frequency (B) and amplitude (C) of endogenous excitatory synaptic events in wild type (n = 27), L-AChR(gf) (n = 19), nlp-12 mutant (n = 21), and nlp-12;L-AChR(gf) (n = 22) animals. Each bar represents mean ± SEM. (PDF) [file pgen.1004584.s005.pdf]

**A**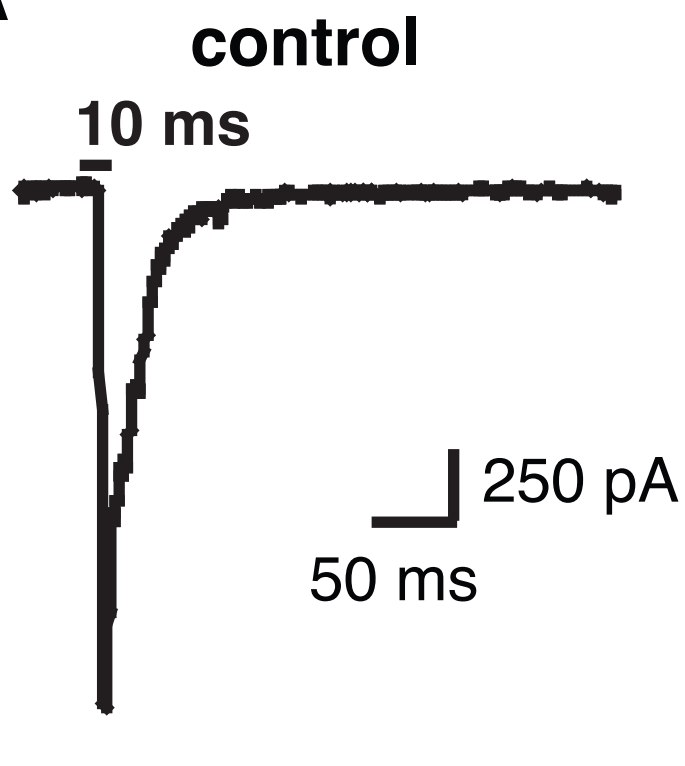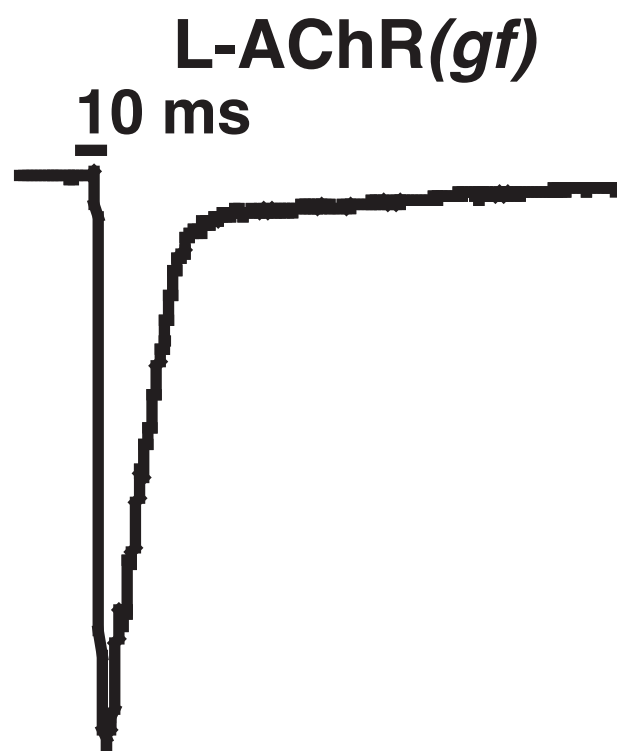**B**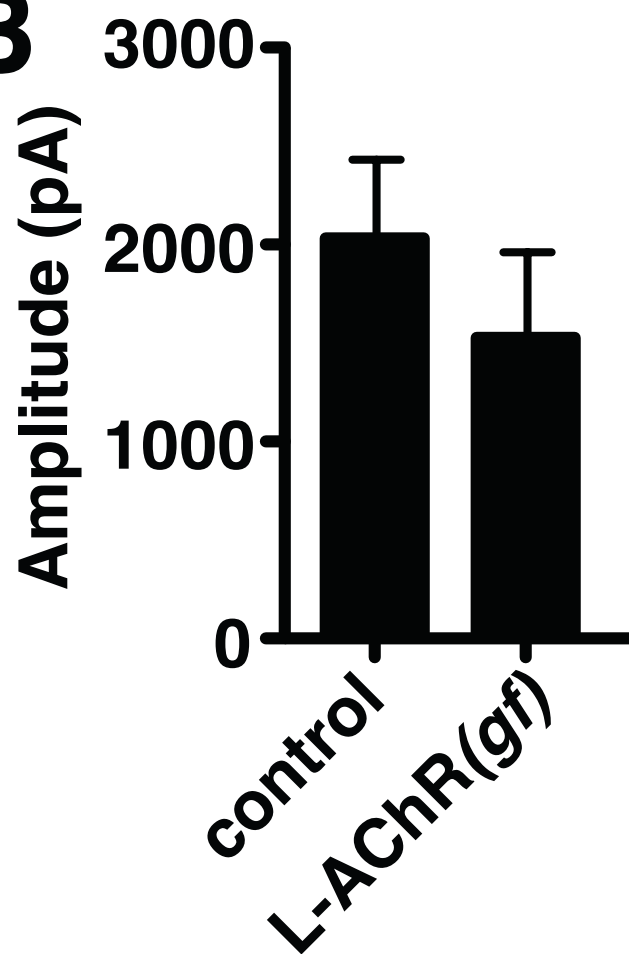**C**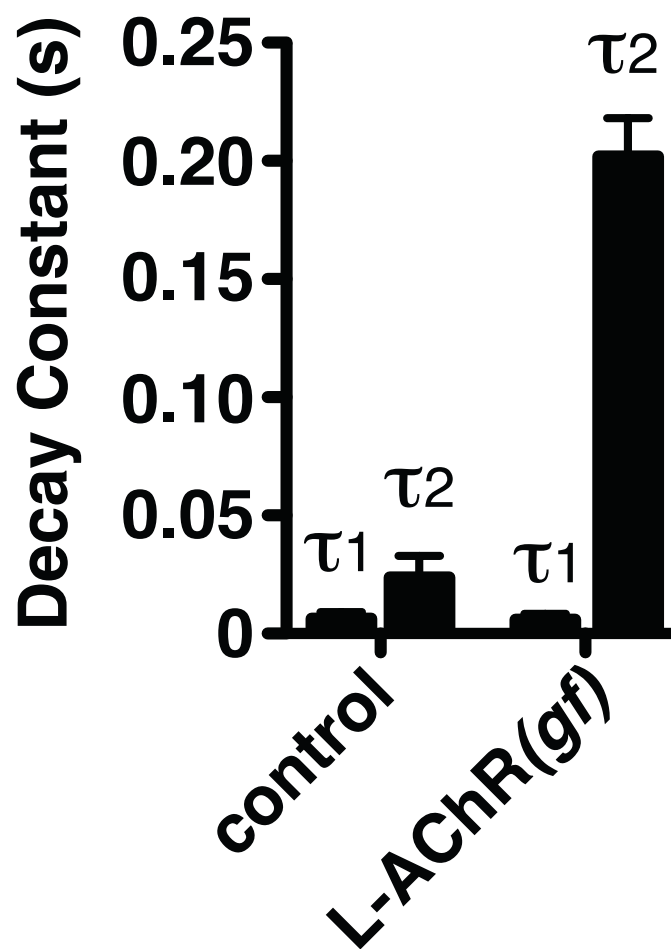

Figure S6

Supplement: Figure S6 — L-AChR(gf) expression prolongs the slow L-AChR mediated component of synaptic currents. (A) Representative muscle current responses to photostimulation of excitatory motor neurons recorded from adult control (n = 10) or L-AChR(gf) (n = 7) animals. Black bar indicates duration of motor neuron photostimulation (10 ms). Holding potential was −80 mV. (B, C) Average amplitude (B) and decay time (C) of photoevoked currents. Currents were fit with two exponentials (τ1 and τ2) to account for slow and fast currents associated with synaptic activation of L-AChR and N-AChR respectively. Both strains stably express the Pacr-2::ChR2-GFP transgene (ufIs23). (PDF) [file pgen.1004584.s006.pdf]
