# Supplementary material for: A Conserved Dopamine-Cholecystokinin Signaling Pathway Shapes Context–Dependent Caenorhabditis elegans Behavior
Source: PLoS Genet. 2014 Aug 28;10(8):e1004584. doi: 10.1371/journal.pgen.1004584 (PMC4148232; doi:10.1371/journal.pgen.1004584)
Supplement: Text S1 — Detailed cloning information for vectors. (PDF) [file pgen.1004584.s007.pdf]

DNA Constructs

***nlp-12* Entry Vectors** (OMF761) 5'-CACCATTATTTTTAAATTTCTATTTTTTTC-3' (forward) and (OMF762) 5'-TTGCCATTCCCAGATTGGCTCGAGC-3' or (OMF860) 5'-CACCTTTGTCGGAGGCAATTGAAATAAGTTT-3' were utilized for generating the *nlp-12* entry clones. CACC was added to the 5' end of primers for directional cloning. The reverse primers were designed either just adjacent to the start codon (*nlp-12* promoter) or at the end of the coding region excluding the stop (*nlp-12* genomic).

Destination Vectors All destination vectors were engineered to contain the *ccd*B reading frame B cassette (Invitrogen, CA).

pDest-17: *ccd*B cassette was SmaI/SphI subcloned into an existing plasmid containing mCherry to generate the destination vector pDest-17 (mCherry).

pDest-27: *ccd*B cassette was MfeI/SphI subcloned into an existing plasmid (pDest-24) containing the trans-splice leader and acceptor sequences followed by mCherry to obtain pDest-27 (SL2::mCherry).

pDest-25 and pDest-34: 720 bp VenusYFP and 1374 bp Tetanus light chain fragments were PCR amplified with 5'-ATTA<u>ACCGGT</u>ATGGTGAGCAAGGGCGAGGA-3' (OMF753), 5'GCTA<u>GCCGGC</u>TTACTTGTACAGCTCGTCCA-3' (OMF754) and 5'-ATTA<u>ACCGGT</u>ATGCCGATCACCATCAACAAC-3' (OMF883), 5'-GCTA<u>GCCGGC</u>TTAAGCGGTACGGTTGTACA-3' (OMF884) primer pairs. The forward and reverse primers contained restriction sites (underlined) for AgeI and NgoMIV, respectively. Digested PCR products were subcloned into AgeI/NgoMIV sites of an existing destination vector (pDest-8) replacing mCherry, to generate pDest-25 (VenusYFP) and pDest-34 (Tetanus toxin light chain).

pDest-60: pCL33 (p*acr-2*::*dop-1*) [51] was digested with BstZ171 and NheI to remove the *acr-2* promoter fragment. *ccd*B cassette was cloned in to generate pDest-60 (*dop-1*).
